# Transcription factor HAT1 is a substrate of SnRK2.3 kinase and negatively regulates ABA synthesis and signaling in Arabidopsis responding to drought

**DOI:** 10.1371/journal.pgen.1007336

**Published:** 2018-04-16

**Authors:** Wenrong Tan, Dawei Zhang, Huapeng Zhou, Ting Zheng, Yanhai Yin, Honghui Lin

**Affiliations:** 1 Key Laboratory of Bio-Resource and Eco-Environment of Ministry of Education, College of Life Sciences, Sichuan University, Chengdu, Sichuan, P.R. China; 2 Department of Genetics, Development, and Cell Biology, Iowa State University, Ames, IA, United States of America; National University of Singapore and Temasek Life Sciences Laboratory, SINGAPORE

## Abstract

Drought is a major threat to plant growth and crop productivity. The phytohormone abscisic acid (ABA) plays a critical role in plant response to drought stress. Although ABA signaling-mediated drought tolerance has been widely investigated in Arabidopsis thaliana, the feedback mechanism and components negatively regulating this pathway are less well understood. Here we identified a member of Arabidopsis HD-ZIP transcription factors HAT1 which can interacts with and be phosphorylated by SnRK2s. *hat1hat3*, loss-of-function mutant of HAT1 and its homolog HAT3, was hypersensitive to ABA in primary root inhibition, ABA-responsive genes expression, and displayed enhanced drought tolerance, whereas *HAT1* overexpressing lines were hyposensitive to ABA and less tolerant to drought stress, suggesting that HAT1 functions as a negative regulator in ABA signaling-mediated drought response. Furthermore, expression levels of ABA biosynthesis genes *ABA3* and *NCED3* were repressed by HAT1 directly binding to their promoters, resulting in the ABA level was increased in *hat1hat3* and reduced in *HAT1OX* lines. Further evidence showed that both protein stability and binding activity of HAT1 was repressed by SnRK2.3 phosphorylation. Overexpressing *SnRK2*.*3* in *HAT1OX* transgenic plant made a reduced HAT1 protein level and suppressed the *HAT1OX* phenotypes in ABA and drought response. Our results thus establish a new negative regulation mechanism of HAT1 which helps plants fine-tune their drought responses.

## Author summary

Drought stress is a key environmental factor that severely reduces crop yield all over the world. The phytohormone abscisic acid (ABA) is known to mediate drought responses through regulating drought-responsive genes expression and stomatal closure, but the mechanisms that negatively regulate this process and prevent the adverse effects of excess drought responses on plant growth is less well studied. Here, we show that a HD-ZIP II transcription factor HAT1 negatively regulates ABA-mediated drought responses through suppressing ABA biosynthesis and signaling. The *hat1hat3* mutant showed ABA-hypersensitive and drought-tolerant phenotypes, whereas the *HAT1*-overexpressing lines were insensitive to ABA and less tolerant to drought. Furthermore, we found SnRK2.3 kinase, a positive component of ABA signaling, interacts with and phosphorylates HAT1 to destabilize and suppress its binding activity. Overexpression of SnRK2.3 reduces HAT1 protein level and inhibits *HAT1OX* phenotypes in ABA and drought responses. Our results revealed a HAT1-mediated negative regulatory mechanism in attenuating the ABA signaling and drought response.

## Introduction

As sessile organisms, plants need to respond and adapt to environmental stress to survive adverse conditions. Plants respond and adapt to stresses through a complex network of factors involved in stress hormone signaling and regulation of gene expression. The phytohormone abscisic acid (ABA) plays a key role in plant responses to biotic and abiotic stress, in particular drought and salinity [1–3].

Since the discovery of ABA receptors, PYRABACTINRESISTANCE1 (PYR1)/PYR1-LIKE (PYL)/REGULATORYCOMPONENTS OF ABA RECEPTORS (RCAR) [4,5], a core ABA signaling pathway has been proposed. In the absence of ABA, group A protein phosphatases type 2C (PP2Cs) interact with subclass III SNF1-related protein kinases (SnRK2.2, 2.3 and 2.6) which keeps the kinases inactive by blocking their catalytic cleft and by dephosphorylating the activation loop [6]. In the presence of ABA, ABA binds to the PYL receptors, forming a PYLs-ABA-PP2C complex and inhibiting phosphatase activity of PP2C [7,8]. This binding and inhibition of the PP2Cs releases the SnRK2s from PP2C-SnRK2 complexes, and the released SnRK2s are activated through autophosphorylation. The activated SnRK2s can then phosphorylate downstream effectors and activate ABA signaling [7,9,10]. Various transcription factors function in ABA signaling-mediated drought response [2,11]. The basic leucine zipper (bzip) family transcription factors including ABF1, ABF2 (AREB1), ABF3, and ABF4 (AREB2), which bind directly to ABREs of stress-responsive genes and stimulate their transcriptional activities, function in the ABA-dependent pathway and are major targets of SnRK2 protein kinases in the ABA core signaling pathway [12–14]. Additionally, some members of the MYB and MYC (bHLH) classes, the No Apical Meristem/Cup-Shaped Cotyledon (NAC), and WRKY families have also been shown to be induced by ABA or abiotic stress or to regulate stress responses, underscoring the importance of transcriptional regulation in plant stress responses [2,15,16]. Transcriptional regulation is one of the most essential mechanisms in the acquisition of stress tolerance [2,17].

However, in many cases, stress adaptation is exchanged for growth and productivity, therefore, it is necessary for plants to develop a resilient system to obtain the optimal trade-off for survival and growth. To this end, plants use elaborate mechanisms associated with posttranscriptional modulation [18] and posttranslational regulation [19,20], as well as transcriptional regulation. In particular, the appropriate control of transcription factors regulating plant growth and development genes is important, because these transcription factors negatively affect plant stress tolerance while being essential for increased productivity. The environmental conditions surrounding plants are constantly changing; thus, posttranslational regulation to control the protein levels of these transcription factors is considered an important mechanism to avoid adverse effects on plant survival. However, the negative components involved in regulation to efficiently coordinate ABA-dependent stress responses are less well known.

The homeodomain-leucine zipper protein (HD-ZIP) family constitute a large family of transcription factors that are unique to plants and is divided into four subfamilies (HD-ZIP I–IV) on the basis of the additional conserved domains, structures and physiological functions [21–23]. HD-ZIP proteins contain homeodomain (HD) that is responsible for specific DNA binding and the closely associated leucine zipper (LZ) domain which acts as a dimerization motif [24]. HD-ZIP proteins can bind to partially inverted repeats such as CAAT(A/T)ATTG (BS1 site), CAAT(C/G)ATTG (BS2 site) or as lightly modified version TAAT(C/T)ATTA for AtHB2/HAT4 [25]. Arabidopsis thaliana homeodomain-leucine zipper protein 1 (HAT1) and its close homologs belong to Class II HD-ZIP of transcription factors that mainly act as repressors by binding to their target genes promoters and play important roles in plant development and in response to the environment [25,26]. Previous works have shown that several members of the family, HAT1, HAT4/AtHB2 and AtHB4, are induced by shade avoidance and overexpression of HAT1 or HAT4 resulted in a similar effect in promoting cell elongation [23,25–27]. HAT2 expression is rapidly induced in response to auxin, and AtHB4 was also reported to modulate auxin, BRs and gibberellin responses [28,29]. It was recently reported that HAT1 is a substrate of BIN2 (BRASSINOSTEROID-INSENSITIVE 2) kinase and appears to function redundantly with other family members such as HAT3 to positively mediate BR responses [30]. HAT1 was also reported to participate in anti-CMV defense response in Arabidopsis and negatively regulates this process [31]. Collectively, these studies indicate that HAT1 is involved in the complex signaling and transcriptional networks coordinating plant growth and stress response. HAT1 promotes plant growth and development by BR signaling or other pathway.

In this study, we demonstrate that HAT1, which was previously reported as a critical regulator in BR-mediated plant growth and in viral defense response, is involved in ABA regulation of drought response by suppressing the ABA biosynthesis and signaling. We found that HAT1 and its homolog HAT3 act redundantly, as the expression of both *HAT1* and *HAT3* were repressed by ABA and drought, and the double mutant *hat1hat3* displayed a reduced ABA sensitivity and enhanced drought tolerance phenotype that was stronger than the single mutants alone. *HAT1*-overexpressing transgenic plants exhibit a hyposensitive response to ABA and drought. Furthermore, we found that HAT1 physically interacts with and can be phosphorylated by SnRK2.3 in vitro and in vivo. SnRK2.3 phosphorylation of HAT1 decreased its protein stability and binding activity. Overexpressing *SnRK2*.*3* in *HAT1OX* transgenic plant can suppress its phenotype in ABA and drought responses. Therefore we identified a new substrate of SnRK2.3 and established a novel negative regulation mechanism by which plants can efficiently coordinate drought responses.

## Results

### HAT1 acts as a negative factor in response to ABA signaling and in osmotic stress tolerance

**Fig 1:**
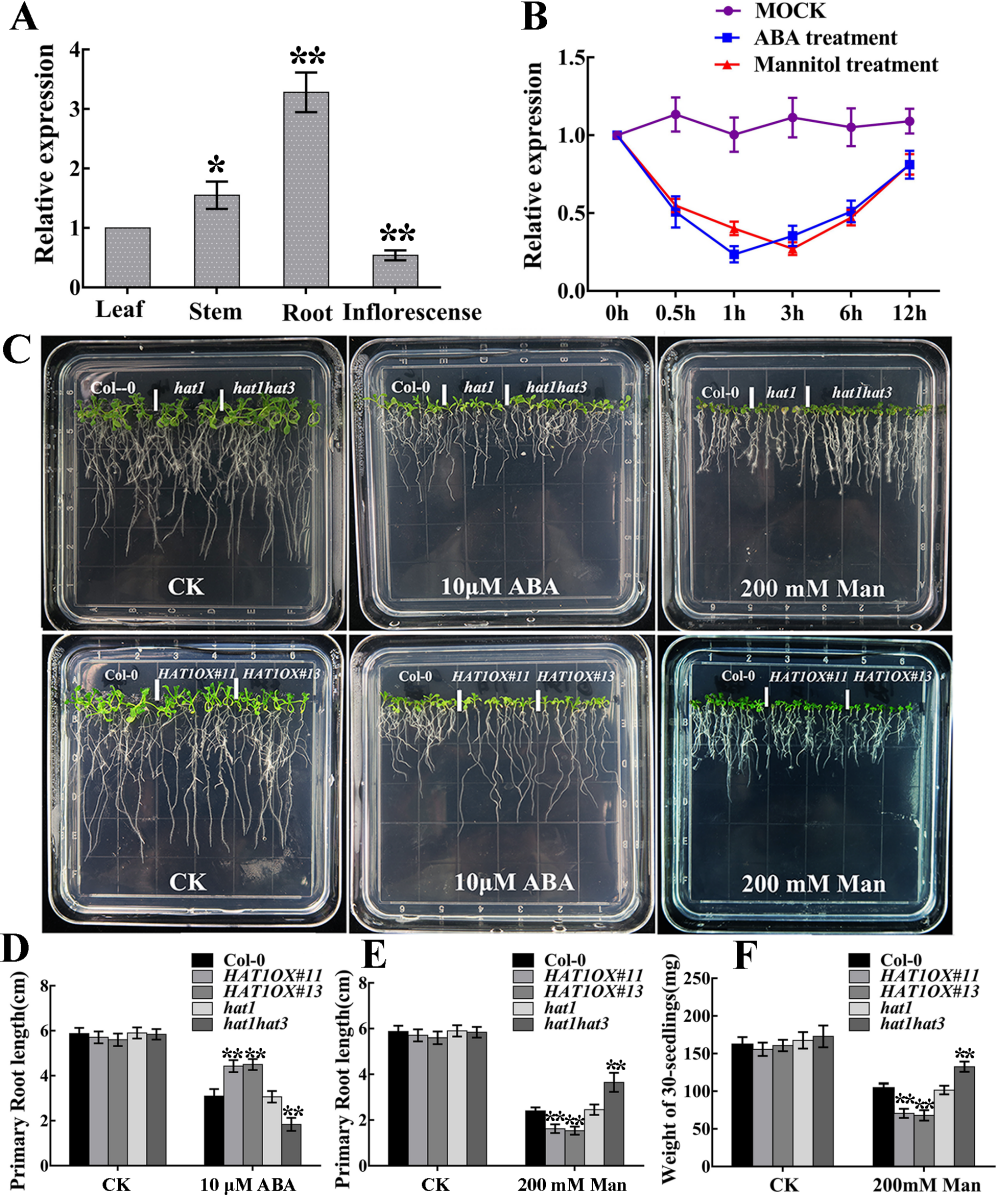
ABA sensitivity and osmotic stress tolerance of *HAT1* mutants (*hat1*, *hat1hat3*) and *HAT1*-overexpressing lines. (A, B) Expression patterns of *HAT1*. (A) qRT-PCR analysis of *HAT1* expression in different tissues. (B) qRT-PCR analysis of *HAT1* expression in response to exogenous ABA and osmotic stress. 12-day-old Col-0 seedlings were transferred to liquid 1/2 MS medium with or without 100 μM ABA or 200 mM mannitol and then the plants were harvested at the indicated time. Data are shown as mean SD of three independent experiments. The significance of difference was analyzed by Student’s t test (*P < 0.05, **P < 0.01). (C) Growth of different genotype seedlings on 1/2 MS medium with or without 10μM ABA or 200 mM mannitol. The 4-day-old seedlings were transferred to 1/2 MS or 1/2 MS medium supplemented 10μM ABA or 200 mM mannitol for 10 days, and then the photos were taken. (D-F) Quantification of primary root length and biomass in different genotypes after ABA treatment or mannitol treatment indicated in (C). CK, control check. Data represent mean ± SD of three independent replicates. asterisks indicate significant differences compared with Col-0 under the same treatment conditions. The significant difference was analyzed by Student’s t test (*P < 0.05, **P < 0.01).

From public data (http://bbc.botany.utoronto.ca/efp/cgi-bin/efpWeb.cgi), we found that *HAT1* expression was reduced after ABA and osmotic stress treatment in seedlings, implying that HAT1 may be implicated in ABA and stress responses. To test the hypothesis, we examined the expression of *HAT1* in different tissues and in seedlings treated with exogenous ABA or osmotic stress. Consistent with the public data, the expression level of *HAT1* was highest in root, and lower in stem, leaf, and inflorescence (Fig 1A), and was significantly repressed by exogenous ABA and osmotic stress (Fig 1B). We further generated GUS reporter lines using HAT1 native promoter and examined the responsiveness of HAT1 expression in the presence of ABA and osmotic stress. As shown in S1A Fig, after ABA and osmotic stress treatments, GUS signals were reduced in cotyledons, leaves and roots as well as guard cells. To determine the subcellular localization of HAT1, we generated constructs that introduced the GFP sequence at the C-terminus of HAT1. The 35S:GFP and 35S:HAT1-GFP constructs were used to transfect Arabidopsis thaliana protoplasts. As shown in S1B Fig, 35S:GFP fluorescence was observed in the entire cell, while HAT1-GFP fusion protein localized in the nucleus. The expression of *HAT3*, its homolog, was similarly regulated, whereas the expression of *HAT2* was not changed by ABA and osmotic stress (S3A and S3B Fig).

To investigate the role of HAT1 in the ABA response and in osmotic stress tolerance, we obtained T-DNA insertion mutants of HAT1, HAT2 and HAT3, *hat1*, *hat2* and *hat3*, respectively. Then we created the double mutant *hat1hat2*, *hat1hat3* and triple mutants *hat1hat2hat3*. The RT-PCR results showed that HAT1 expression was hardly detected in *hat1*, *hat1hat2*, *hat1hat3* and *hat1hat2hat3* mutants. Similarly, transcript of HAT2 was not observed in *hat2*, *hat1hat2* and *hat1hat2hat3* mutants, and HAT3 transcript was abolished in *hat3*, *hat1hat3* or triple (*hat1hat2hat3*) mutants (S2A Fig). Western blotting using an anti-GFP antibody showed that HAT1-GFP accumulated in the two *HAT1OX* lines (S2B Fig). Next, we analyzed ABA sensitivity with regard to seedlings growth in Col-0, *HAT1OX* lines and knockout mutants. The 4-day-old seedlings grown on 1/2 MS medium were transferred to 10 μM ABA-containing medium for 10 days. As shown in Fig 1C and 1D and S3C and S3D Fig, root growth of double mutant *hat1hat3* or triple mutant *hat1hat2hat3* was dramatically retarded under ABA conditions compared with that of wide-type *Columbia-0* (Col-0) and was similar among Col-0, *hat1*, *hat2*, *hat3* and *hat1hat2* with or without ABA treatment. To analyze the function of HAT1 in osmotic stress tolerance, 4-day-old Col-0 and knockout mutants were treated with mannitol, a stress treatment commonly used to mimic osmotic stress tolerance in the laboratory. The double mutant *hat1hat3* or triple mutant *hat1hat2hat3* displayed less inhibition on growth in the medium containing mannitol compared with Col-0, while the single mutant *hat1*, *hat2*, *hat3* and double mutant *hat1hat2* showed little difference after mannitol treatment in comparison with Col-0 (Fig 1C, 1E and 1F and S3C, S3E and S3F Fig). In contrast, the two *HAT1*-overexpressing lines (*HAT1OX#11* and *HAT1OX#13*) showed significantly reduced ABA sensitivity and osmotic stress tolerance (Fig 1C bottom, Fig 1D, 1E and 1F). Together, these data indicate that HAT1 plays a negative role in ABA signaling and in osmotic stress tolerance, and it is functionally redundant with HAT3 in ABA and osmotic stress response.

### HAT1 impairs ABA-induced stomatal closure and drought tolerance

**Fig 2:**
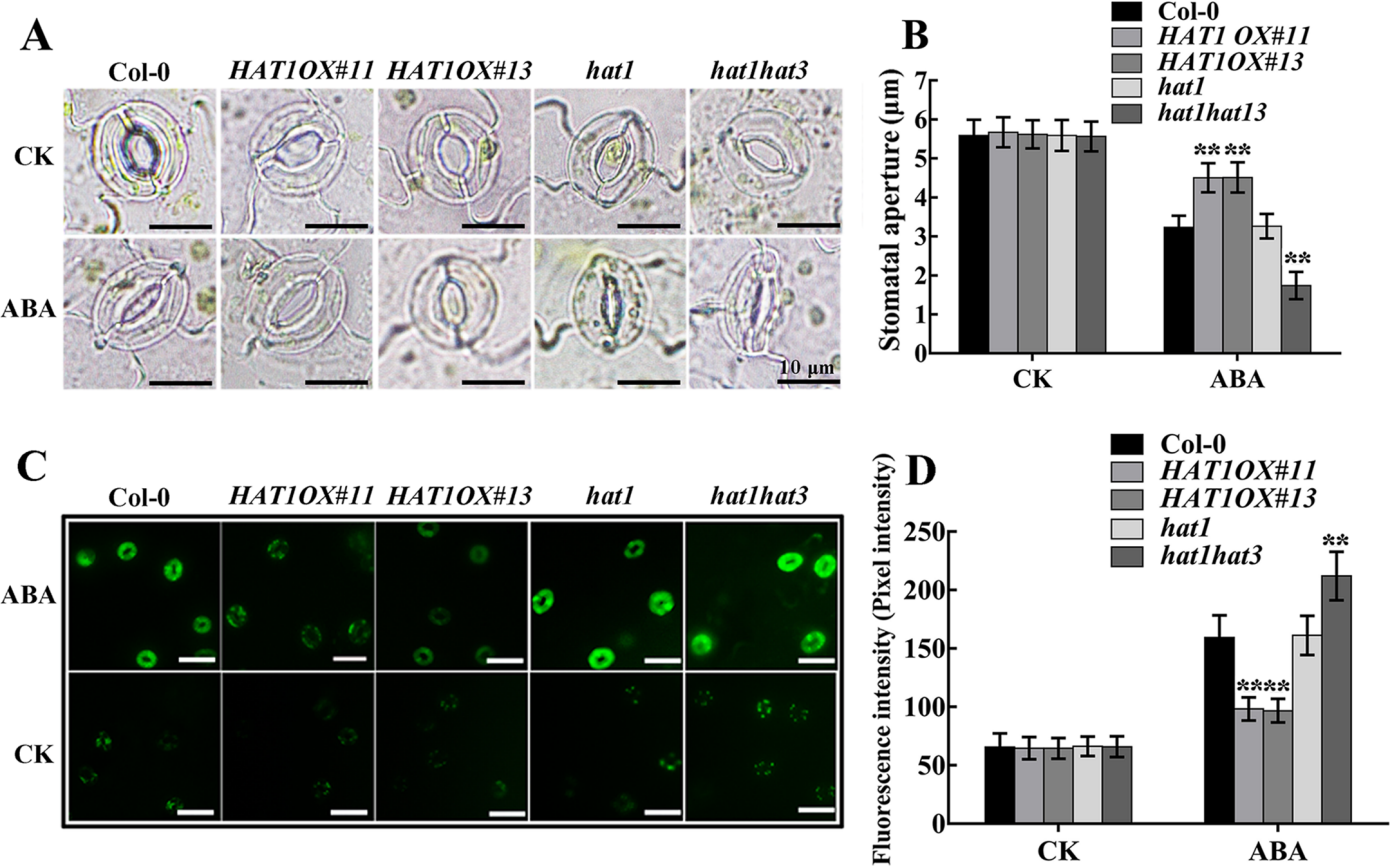
HAT1 suppresses ABA-induced stomatal closure and H_2_O_2_ accumulation in guard cells. (A) Epidermal peels of indicated genotypes were treated with or without ABA for 3 h after stomatal pre-opening under light for 2h, and the stomatal aperture was measured. Scale bars: 10 μm. (B) Stomatal apertures of different genotypes indicated in (A). (C, D) Fluorescence images (C) and pixel intensities (D) in guard cells preloaded with 50 μM H2DCFDA for 10 min in darkness. Each assay was repeated at least three times. The data are presented as means ± SD. (Student’s t-test: ** P<0.01). Scale bar: 25 μm.

**Fig 3:**
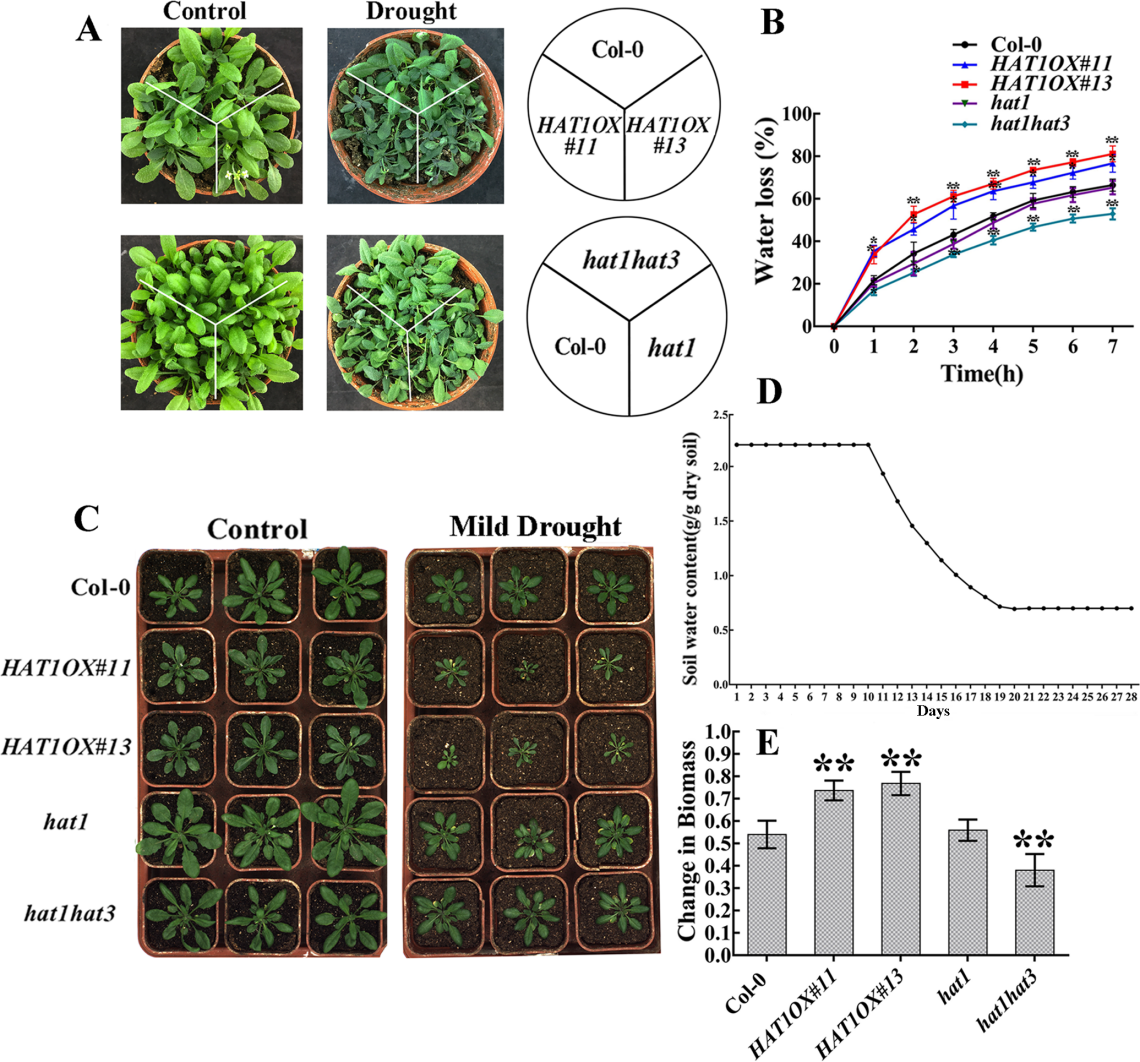
HAT1 negatively regulate drought stress responses in Arabidopsis thaliana. (A) The phenotypes of Col-0, HAT1OX lines, *hat* mutants (*hat1*, *hat1hat3*) in response to progressive drought stress. Different genotype plants were grown in soil with sufficient water for 3 weeks (Watered), and then water was withheld for 10 d (Progressive drought), and the photos were taken. (B) Water loss from detached leaves of different genotype plants. Leaves at similar developmental stages were excised and weighed at the indicated time after detachment. The proportion of fresh weight losses was calculated on the basis of the initial weight of the leaves. Data are shown as mean SD of three independent experiments. The significance of difference was analyzed by Student’s t test (**P < 0.01). (C-E) Growth of Col-0, *HAT1OX* lines, *hat1* mutants (*hat1*, *hat1hat3*) in response to mild drought stress. 3-week-old plants were subjected to mild drought treatment and the images of both drought-treated plants (right) and the well-watered plants (left) were taken (C). (D) Water loss from the peat pellets during the duration of the experiment. Control soil water content was maintained at a constant value of 2.2 g water g^-1^ dry soil (solid line) during the entire experiment. For the mild drought condition, soil water content was maintained at 0.7g H_2_O g^-1^ dry soil. (E) The change in biomass under mild drought among different genotypes compared to Col-0. After mild drought treatment, all the replications of the drought-treated and the well-watered control were harvested and the dry weights (biomass) were measured. Then calculate the reduction in biomass. Bars indicate SD calculated from three independent experiments. The significance of difference was analyzed by Student’s t test and Asterisks indicate significant difference from the wild type (*P < 0.05, **P < 0.01).

ABA regulation of stomatal movements is a well established model system for the study of plants response to drought stress. Thus, we measured the stomatal aperture from epidermal peels of Col-0, *HAT1OX* lines and knockout mutants. Overexpression of *HAT1* suppressed ABA-mediated stomatal closure, while double mutant *hat1hat3* and triple mutant *hat1hat2hat3* exhibited an accelerated ABA sensitivity in stomatal closure and single mutants(*hat1*, *hat2*, *hat3*) or *hat1hat2* showed little difference after ABA treatment in comparison with Col-0 (Fig 2A and 2B and S4A and S4B Fig), indicating that HAT1 and HAT3 function redundantly in regulating ABA-mediated stomatal closure. As H_2_O_2_ acts as an important signal molecular in ABA-induced stomatal closure, H_2_O_2_ accumulation in guard cells was measured by a fluorescence dye, 2,7-dichlorodihydro fluorescein diacetate (H2DCF-DA) [32,33]. As shown in Fig 2C and 2D, H_2_O_2_ accumulation in guard cells was less in *HAT1OX* lines, more in *hat1hat3* double mutant, compared to Col-0 and *hat1* after ABA treatment, suggesting that HAT1-impaired stomatal closure may be caused by changed H_2_O_2_ in guard cells. Next, we tested whether HAT1 plays a role in the drought stress response. When exposed to dehydration stress by withholding water for 10 days, *HAT1OX* lines displayed a withered phenotype, while the *hat1hat3* double mutant largely remained turgid and single mutant *hat1* showed little difference in comparison with Col-0 (Fig 3A). Measurement of leaves water loss showed that *HAT1OX* lines lost water much faster, while *hat1hat3* displayed reduced water-loss rate than Col-0 and *hat1* (Fig 3B). As a result, overexpression of HAT1 markly reduced plant survival under drought stress, whereas *hat1hat3* showed enhanced survival compared to Col-0 and *hat1* (S5A–S5C Fig).

To study the responses of different genotypes to controlled soil water deficit drought, Col-0, *HAT1OX* lines and knock-out mutants (*hat1*, *hat1hat3*) were grown for 3 weeks under well-water condition (2.2g H_2_O/g dry soil) and then subjected to mild drought stress (Fig 3D). After grown under mild drought condition (0.7g H_2_O/g dry soil) for 9days, the biomass of both drought-treated and well-watered plants was measured and then the change in biomass was calculated. As shown in Fig 3C and 3E, *HAT1OX* lines showed more reduction in biomass compared to the Col-0 which is considered drought sensitive genotype, while the double mutant *hat1hat3* displayed less reduction in biomass and *hat1* showed similar reduction in biomass in comparison to Col-0. Altogether, these data demonstrate that HAT1 and HAT3 function redundantly and negatively to regulate ABA-mediated stomatal closure and drought response.

### HAT1 negatively regulates drought-responsive genes and positively regulates *PP2Cs*

**Fig 4:**
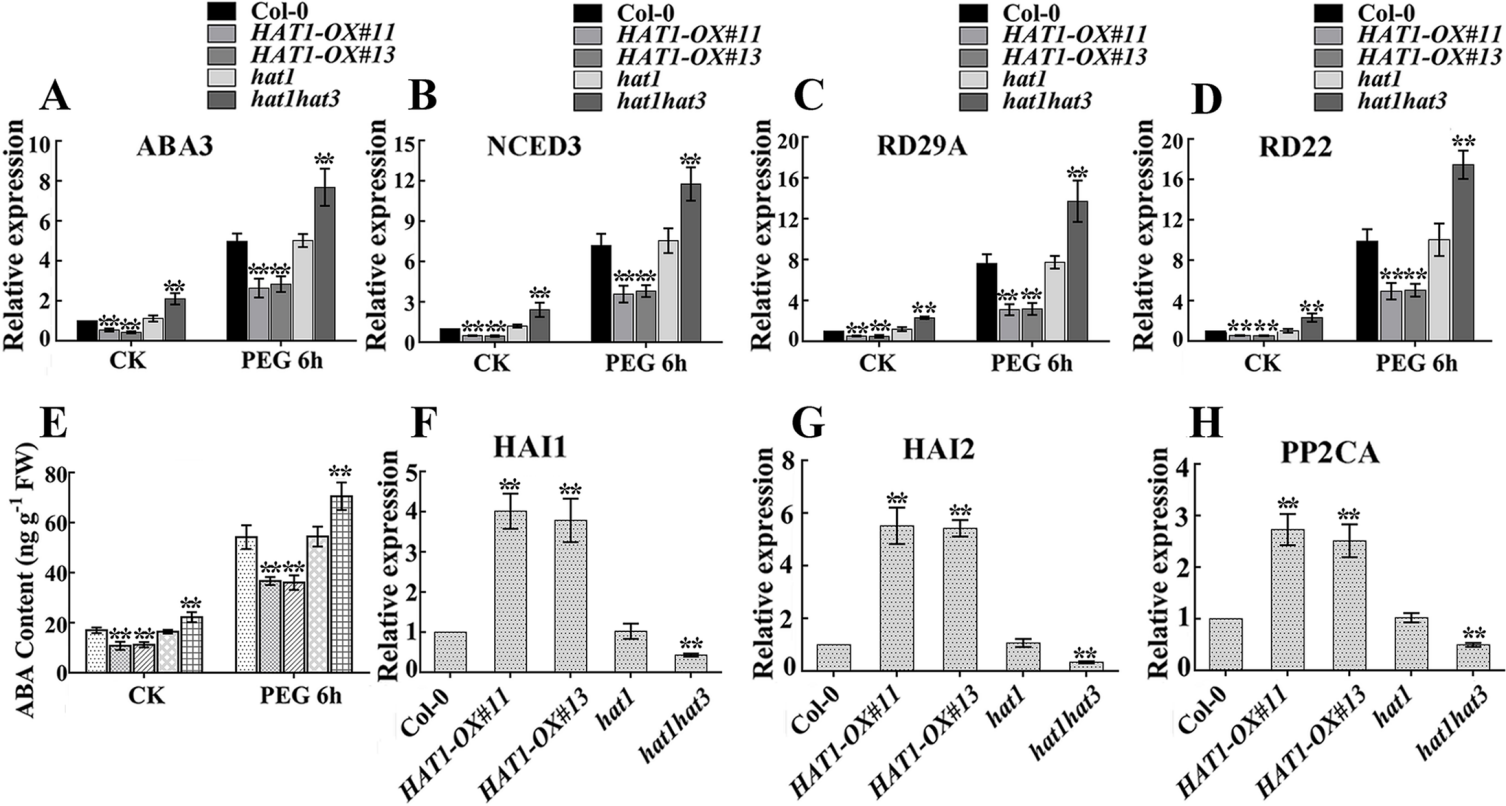
Expression profiles of ABA or drought stress-responsive genes. (A-D) qRT-PCR analysis of *ABA3* (A), *NCED3* (B), *RD29A* (C), and *RD22* (D) expression. 12-day-old seedlings were transferred to liquid MS medium with or without 15% PEG6000 and sampled after 6h treatment. Data are means of three replicates ± SD, asterisks indicate significant differences compared with Col-0 under the same treatment conditions. The significance of difference was analyzed by Student’s t test (*P < 0.05, **P < 0.01). (E) ABA content as determined by ELISA. Data are shown as mean SD of three independent experiments. The significance of difference was analyzed by Student’s t test (*P < 0.05, **P < 0.01). (F-H) qRT-PCR analysis of PP2Cs expression, *HAI1* in (F), *HAI2* in (G), *PP2CA* in (H). Three independent repeats were performed, the significance of difference was analyzed by Student’s t test (*P < 0.05, **P < 0.01).

As HAT1 is a negative regulator of ABA signaling and drought response (Fig 1 and Fig 2), the expression of ABA or drought stress inducible marker genes were tested in different genotypes. We first determined the transcript levels of ABA response maker genes which were also ABA biosynthesis genes. These genes include *ABA1* [34], *AAO3* [35], *ABA3* [36], and *NCED3* [37]. Among the four genes, the expression of *ABA3* and *NCED3* were significantly reduced in *HAT1OX* lines and up-regulated in *hat1hat3* double mutant under both control and osmotic stress conditions (Fig 4A and 4B). To determine whether or not ABA levels were affected, we quantified the ABA content in different genotypes. Under normal conditions, ABA level in *HAT1OX* seedlings was found to be lower than that in Col-0 and *hat1* single mutants, whereas it was elevated in *hat1hat3* double mutant. When exposed to 15% polyethylene glycol (PEG) 6000 that mimics a drought stress, *HAT1OX lines* had a reduced ABA level, and the *hat1hat3* double mutants accumulated higher level of ABA compared with Col-0 and *hat1* (Fig 4E). In addition, the induction of *RD29A* and *RD22*, which are well established drought-induced marker genes [16,38], was also tested in different genotypes. As expected, the expression of these two genes were reduced in *HAT1OX* lines and elevated in *hat1hat3* double mutant compared with Col-0 and *hat1* (Fig 4C and 4D). Furthermore, the expression of *HAI1*, *HAI2* and *PP2CA*, which belong to PP2Cs, negative regulators of ABA signaling, was diminished by down-regulation of both *HAT1* and *HAT3* and enhanced by *HAT1* overexpression (Fig 4F–4H). Taken together, these results indicate that HAT1 repressed drought-responsive genes and induced *PP2C* genes, which may account for the repressed drought tolerance in *HAT1OX* lines.

### HAT1 physically interacts with SnRK2.2/2.3/2.6 and can be phosphorylated by SnRK2.3

**Fig 5:**
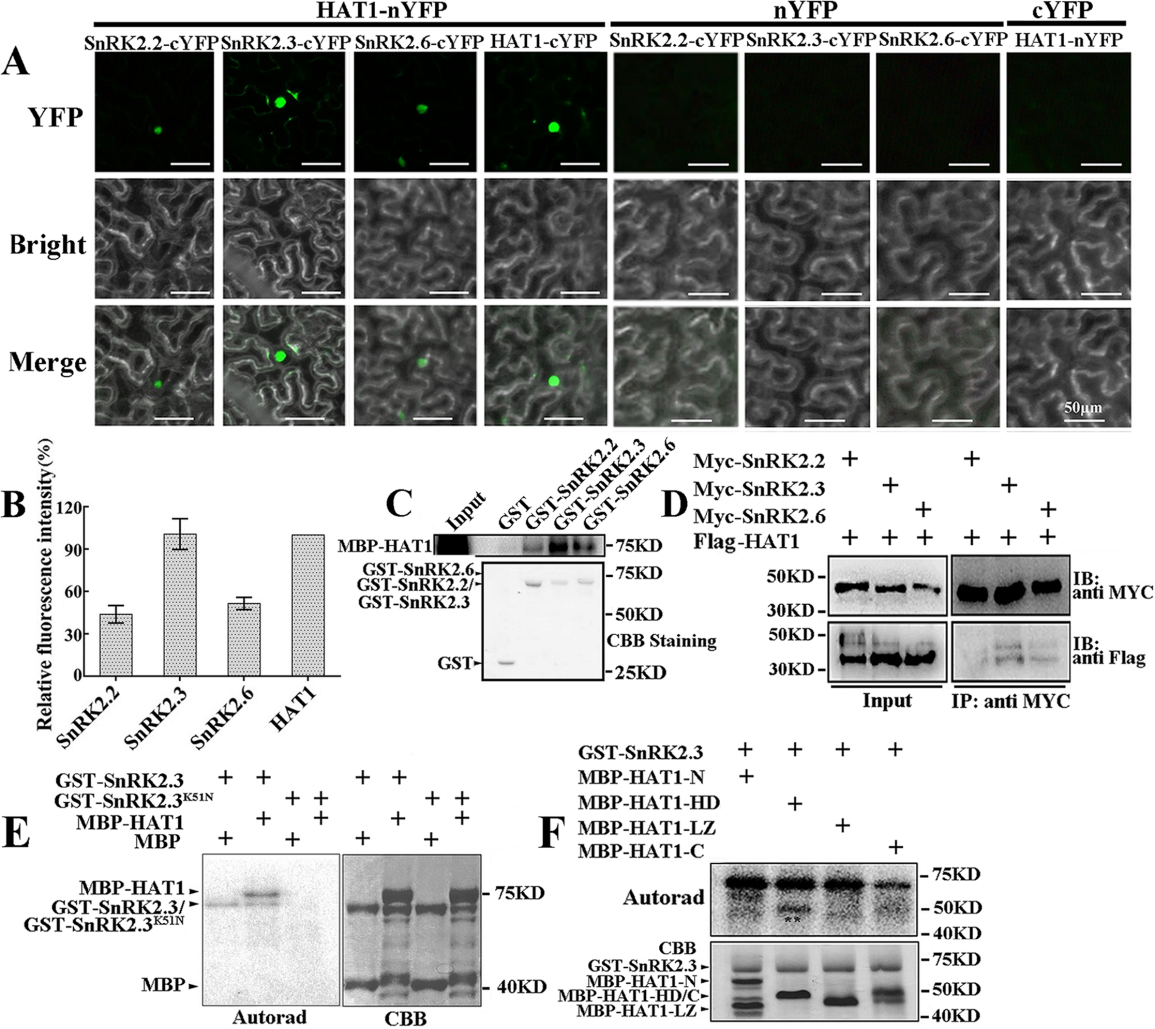
SnRK2.3 interacts with and phosphorylates HAT1. BiFC assays of interactions of HAT1 with the indicated SnRKs in N. benthamiana leaves (A) and quantification of relative fluorescence intensities (relative to that of HAT1-HAT1 interaction) in BiFC analyses (B). HAT1-nYFP/ HAT1-cYFP were used as positive control and nYFP/HAT1-cYFP and cYFP/SnRK2.2/ SnRK2.3/ SnRK2.6 were used as negative controls. Data represent mean ± SD of three independent replicates. Ten cells were analyzed in each replicate for each construct combination. Scale bars: 50 μm. Images were acquired using identical settings. (C) SnRK2s interacts with HAT1 in GST pull-down assay. GST, GST-SnRK2s and MBP-tagged HAT1 were used in this assay. HAT1 was detected by western blotting with anti-MBP antibody. (D) CoIP analysis of the interaction between SnRK2s and HAT1 in Arabidopsis leaf protoplasts. Myc-fused SnRKs (SnRK2.2/2.3/2.6) were immunoprecipitated using anti-Myc beads, and coimmunoprecipitated HAT1-Flag was then detected using an anti-Flag antibody. Experimental details are provided in Methods. (E, F) SnRK2.3 phosphorylates HAT1 in vitro. GST-SnRK2.3 was used to phosphorylate MBP or MBP-HAT1 (E) or truncated MBP-HAT1 (F).

The SnRK2 kinases are integral positive component of ABA signaling, and phosphorylate S/T residues in the RXXS/T motif in their substrates. There are 7 potential phosphorylation sites for SnRK2 kinases in the predicted HAT1 protein, which prompted us to test whether HAT1 was a substrate of SnRK2 kinases. First, we tested if subclass III SnRK2s could physically interact with HAT1. Bimolecular fluorescence complementation (BiFC) analysis was performed to examine the interaction of HAT1 with SnRK2.2, SnRK2.3, and SnRK2.6 in plants. We found that HAT1 interacts with all subgroup III SnRK2s in the nucleus and no fluorescence signal was detected in the negative controls (Fig 5A). Quantitative analyses of BiFC signals showed strong SnRK2.3-HAT1 interactions and weak signals for HAT1 interaction with other subgroup III SnRK2s (Fig 5B). GST pull-down experiment confirmed this interaction in vitro (Fig 5C). GST-SnRK2s, but not GST alone, pulled down a significant amount of MBP-HAT1 protein, demonstrating a direct interaction between SnRK2s and HAT1. Consistent with the result of BiFC assays, the interaction between SnRK2.3 and HAT1 is the strongest (Fig 5C). The in vivo interaction of SnRK2s with HAT1 were corroborated by co-immunoprecipitation (Co-IP) assay using Arabidopsis protoplasts co-expressing Myc-SnRK2s and HAT1-Flag fusion constructs (Fig 5D). We also generated a series of truncated HAT1 fragments (HAT1-1F (135–282), HAT1-2F (192–282), HAT1-3F (234–282)) which were fused with the C-terminal half of YFP and transformed them individually with SnRK2.3-nYFP into tobacco leaves. When deleted to amino acid 134 in HAT1, only a weak fluorescent signal was detected, while deletions to amino acid 191 and 233 in HAT1 totally abolished the interaction with SnRK2.3 (S6B Fig). Several truncated MBP-HAT1 (N-terminal region, HD, LZ, and C-terminal region of HAT1) were further used to map the specific domain of HAT1 required for the interaction with SnRK2.3. As shown in S6C Fig, HAT1 interacts with Snrk2.3 with its N-terminal region. Taken together, the N-terminal region in HAT1 mediates the interaction between HAT1 and SnRK2.3. Further, we conducted in vitro kinase assays to test whether SnRK2.3 can phosphorylate MBP-fusion HAT1 protein and found that SnRK2.3 can phosphorylate HAT1, but not MBP (Fig 5E). The kinase dead form of SnRK2.3 (SnRK2.3^K51N^) was used as a negative control and it totally abolished the phosphorylation of SnRK2.3 on HAT1 (Fig 5E). We further found that the homeodomain of HAT1 (MBP-HAT1-HD) can be phosphorylated by SnRK2.3 rather than the other regions (Fig 5F). In addition, the interaction of SnRK2.3 with HAT3 was also examined by BiFC analysis. As shown in S7A Fig, HAT3 interacts with SnRK2.3 in the nucleus, suggesting that SnRK2.3 may regulate HAT3 through a similar manner as HAT1.

### Proteasome-mediated HAT1 degradation is triggered by SnRK2.3 phosphorylation

**Fig 6:**
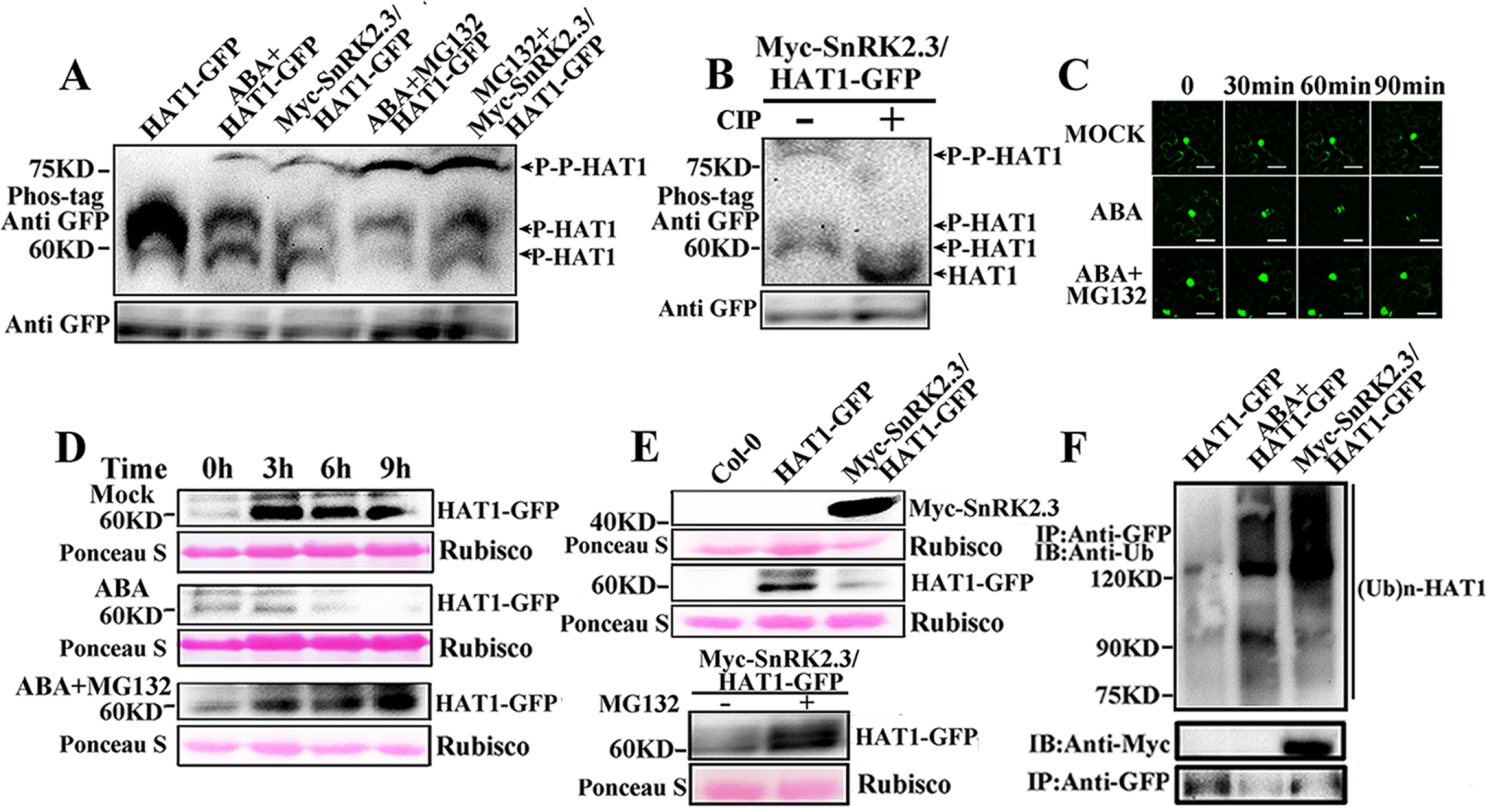
ABA treatment and SnRK2.3 phosphorylation destabilizes HAT1. (A) SnRK2.3 phosphorylates HAT1 in vivo. HAT-GFP was prepared from *HAT1OX* transgenic plants treated with or without ABA or ABA plus MG132 or from *SnRK2*.*3OX/HAT1OX* transgenic plants treated with or without MG132, and then separated on SDS/PAGE gel containing Phos-tag reagent (NARD Institute). (B) Characterization of up-shifted band of HAT1 by dephosphorylation. HAT1 immunoprecipitated from *SnRK2*.*3OX/HAT1OX* transgenic plants with anti GFP beads were incubated with or without calf intestinal alkaline phosphatase (CIP) (sigma) and then separated on Phos-tag SDS-PAGE. The HAT1-GFP protein was detected by immunoblot analysis using anti-GFP antibody. (C) Time microscope images of a representative *Nicotianabenthamiana* leaf epidermal cell expressing HAT1-GFP exposed to 50 μM ABA. Results are representative microscope images from three separate experiments, and at least 5 independent cell images were captured per experiment. Scale bars: 50 μm. (D) ABA promotes HAT1 degradation through the proteasome. 12-day-old seedlings grown one half-strength MS plates were treated with (+) or without (-) 50μM ABA and/or 30μM MG132 for indicated time period. Total protein was extracted and analyzed by immunoblotting. Ponceau S staining was used to demonstrate equal loading. (E) SnRK2.3 promotes the degradation of HAT1 in vivo. Proteins extracts from 12-day-old *HAT1OX* seedlings or from *SnRK2*.*3OX/HAT1OX* seedlings treated with or without MG132 were subjected to immunoblot analysis using anti-GFP for HAT1 and anti-Myc antibody for SnRK2.3. (F) Ubiquitination level of HAT1 is enhanced by SnRK2.3 in vivo. HAT1-GFP protein was immunoprecipitated from 12-day-old *HAT1OX* seedlings treated with or without ABA and in *SnRK2*.*3OX/HAT1OX* seedlings. Immunoprecipitated proteins were detected by immunoblotting using anti-GFP or anti-Ubiquitin (Ub) antibody (Abcam) for HAT1 and anti-Myc antibody for SnRK2.3.

To test whether phosphorylation of HAT1 by SnRK2.3 in vivo, the HAT1-GFP was immunoprecipitated from *HAT1OX* or *SnRK2*.*3OX/HAT1OX* transgenic seedlings treated with/without ABA or MG132 and detect the phosphorylation/dephosphorylation form using phos-tag gel blot analysis with an anti-GFP antibody (Fig 6A). Two faster-migrating bands can be detected in untreated plants. We found that ABA treatment or *SnRK2*.*3* overexpression resulted in the appearance of a slower-migrating HAT1 in *HATOX* transgenic plants (Fig 6A). When subjected to phosphatase [calf-intestinal alkaline phosphatase (CIP)] treatment, all three bands disappeared and a new lower band which is likely the unphosphorylated form of HAT1 appeared, indicating that HAT1 exists mostly as phosphorylated forms in plants and an elevated phosphorylation of HAT1 is formed by ABA treatment or *SnRK2*.*3* overexpression (Fig 6A and 6B). Furthermore, when treated with MG132, the phosphorylation level of HAT1 was significantly increased in ABA-treated *HAT1OX* or *SnRK2*.*3OX/HAT1OX* seedlings, indicating that super-phosphorylation form of HAT1 was instable (Fig 6A).

To investigate the function of the SnRK2.3 phosphorylation on HAT1 protein stability, we detect HAT1-GFP protein level in transgenic plants. First, we expressed HAT1-GFP fusion proteins in *Nicotiana benthamiana* epidermal cells and examined the effects of ABA and the proteasome inhibitor MG132 on GFP fluorescence. Time-course microscopic observation revealed that the HAT1-GFP fluorescence intensity was substantially reduced in leaves treated with ABA alone, whereas HAT1-GFP was more stable after application of ABA plus MG132 (Fig 6C). Similarly, HAT3-GFP fluorescence intensity was also rapidly reduced in response to ABA treatment and only slightly altered in response to the control stimulus (solvent used for ABA) and combined ABA and MG132 (S7B Fig). We then examined HAT1-GFP protein level in HAT1-GFP transgenic plants. As shown in Fig 6D, HAT1 protein increased in the liquid one-half MS (Murashige and Skoog) medium without ABA treatment (Fig 6D top panel). However, in the presence of ABA, HAT1-GFP protein clearly decreased in relation to the mock treatment after 3 h of treatment (Fig 6D middle panel). When we treated plants with ABA and MG132 together, the HAT1 protein level significantly increased as mock (Fig 6D bottom panel), suggesting that ABA triggers proteasome-mediated HAT1 degradation.

To investigate whether ABA-induced HAT1 degradation is mediated by SnRK2.3 phosphorylation in plant, we detected HAT1-GFP protein level in *HAT1OX* or *SnRK2*.*3OX/HAT1OX* transgenic seedlings. The transcriptional level of HAT1 was same in *HAT1OX* and *SnRK2*.*3OX/HAT1OX* (S8 Fig). As shown in Fig 6E, HAT1 protein was clearly degraded in *SnRK2*.*3OX/HAT1OX* transgenic plants, while this degradation was blocked by addition of MG132. We further examined the ubiquitination level of HAT1 in ABA-treated *HAT1OX* and in *SnRK2*.*3OX/HAT1OX* transgenic plants. As shown in Fig 6F, the ubiquitinated level of HAT1 was significantly increased in *HAT1OX* plants after treatment with ABA, or in *SnRK2*.*3OX/HAT1OX* transgenic plants. Taken together, these results indicated that SnRK2.3-mediated HAT1 phosphorylation facilitates the degradation of HAT1 via stimulating its ubiquitination.

### SnRK2.3 represses the binding ability of HAT1 to HB site on promoter of *ABA3* and *NCED3*

**Fig 7:**
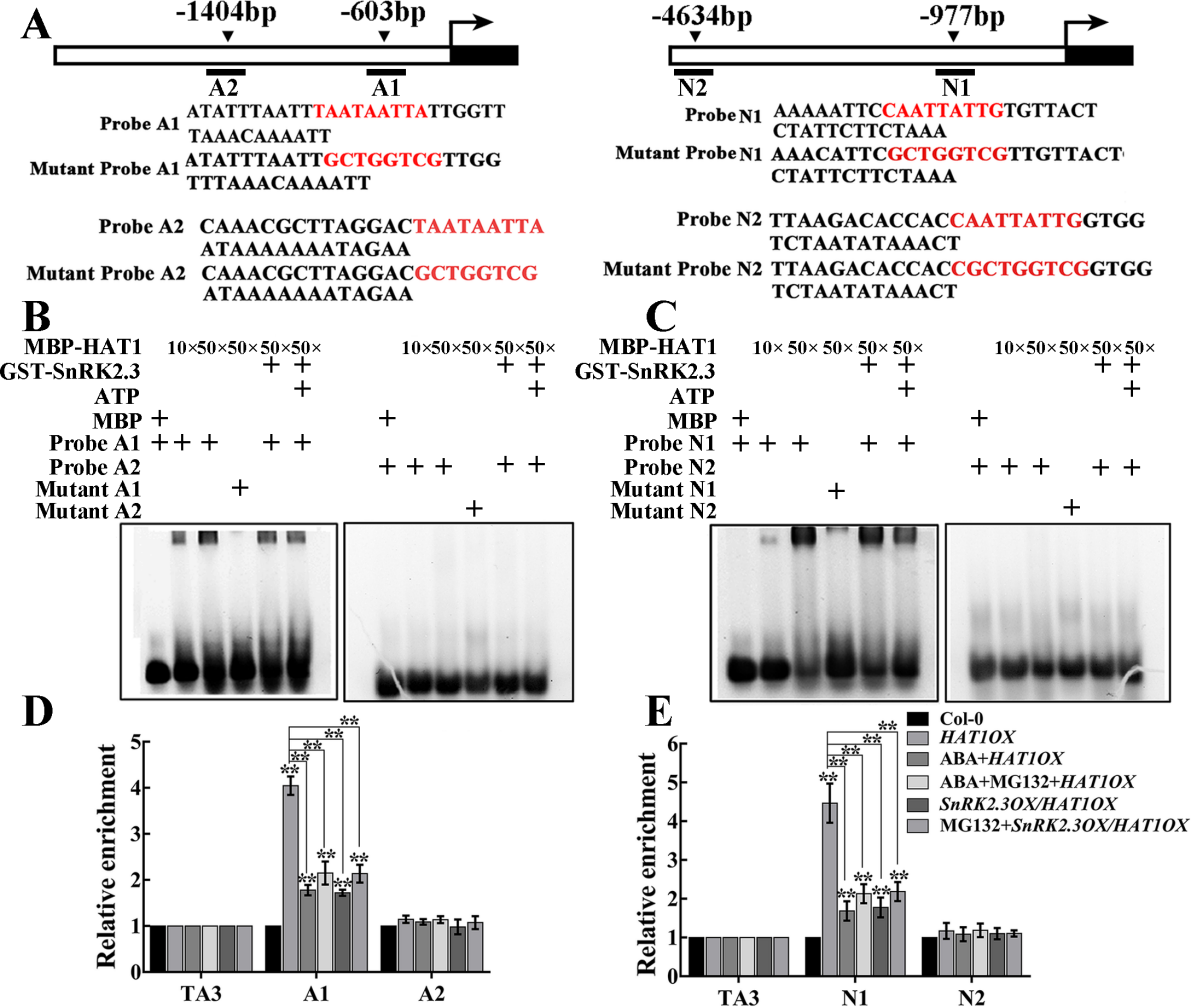
SnRK2.3 phosphorylation represses the binding ability of HAT1 to the promoters of *ABA3* and *NCED3*. (A) Schematic representation of *ABA3* and *NCED3* promoter. The upstream region and part of an exon of *ABA3* and *NCED3* are shown with a white box and black box, respectively. The arrowheads in the top indicate the sites containing HB sites in the *ABA3* and *NCED3* promoter. Black lines represent the DNA fragments amplified in the ChIP assay. Sequences of wild-type probe with HB sites and various mutant probes were shown. (B, C) Electrophoretic mobility shift assays (EMSAs) to examine HAT1 binding to the *ABA3* (B) and *NCED3* (C) promoter. MBP-HAT1 was immunoprecipitated with MBP agarose and incubated with purified GST-SnRK2.3 with kinase reaction buffer at 37°C for 30 min. The MBP, MBP-HAT1, phosphorylated MBP-HAT1 and GST-SnRK2.3 proteins were incubated with the WT or mutants probes. (D, E) ChIP-qPCR assay of HAT1 binding to *ABA3* (D) and *NCED3* (E) promoter in vivo. The 12-day-old *HAT1OX* were treated with 50 μM ABA or ABA plus MG132 for 3h, and the *SnRK2*.*3OX/HAT1OX* seedlings were treated with/without 30 μM MG132 alone for 3h, then the seedlings were harvested for ChIP-qPCR assay using anti-GFP antibody. Data are shown as mean SD of three independent experiments. The significance of difference was analyzed by Student’s t test (*P < 0.05, **P < 0.01).

HAT1 acts as a regulator by binding to HB site within its target genes promoters. First, we analyzed promoter sequences of four ABA or drought-responsive genes (*ABA3*, *NCED3*, *RD29A*, *RD22*) and found that there were two HB-binding sites within the *ABA3* and *NCED3* promoter regions respectively (Fig 7A). To determine whether or not HAT1 bind to the *ABA3* and *NCED3* promoter, electrophoresis mobility shift assays (EMSAs) were conducted. The MBP-HAT1 fusion protein can bind to A1 fragment of *ABA3* promoter and N1 fragment of *NCED3* promoter, but this binding was abolished by mutation of HB sites in the probes (Fig 7B and 7C). The addition of GST-SnRK2.3 fusion protein was able to slightly inhibit the ability of HAT1 binding to the A1 fragment and N1 fragment (Fig 7B and 7C). When HAT1 was phosphorylated by SnRK2.3 in vitro, the binding affinity of phosphorylated HAT1 was dramatically reduced (Fig 7B and 7C). These data indicate that HAT1 protein can bind to the A1 fragment of *ABA3* promoter and N1 fragment of *NCED3* in vitro, and its binding ability is repressed by SnRK2.3 phosphorylation.

To further test the effect of SnRK2.3 on the binding ability of HAT1 in vivo, we performed chromatin immunoprecipitation (ChIP) assays. We immunoprecipited HAT1-GFP protein from *HAT1OX* transgenic seedlings treated with/without ABA or ABA in combination with MG132 with anti-GFP antibody. TA3, a retrotransposable element, was used as the internal control [39]. ChIP-qPCR results indicated that HAT1 specifically bound to the A1 region of ABA3 and N1 region of NCED3, and other genomic fragments containing HB sites were not targeted by HAT1 (Fig 7D and 7E). The binding ability of HAT1 was reduced by both ABA treatment and ABA plus MG132 treatment (Fig 7D and 7E). Furthermore, HAT1 binding ability was significantly diminished by SnRK2.3 overexpression, and it cannot be recovered by addition of MG132 (Fig 7D and 7E). Altogether, these results support that SnRK2.3 represses the binding ability of HAT1 by phosphorylation.

### *SnRK2*.*3* Overexpression suppresses ABA-insensitivity and drought-hypersensitivity of *HAT1OX* plants

**Fig 8:**
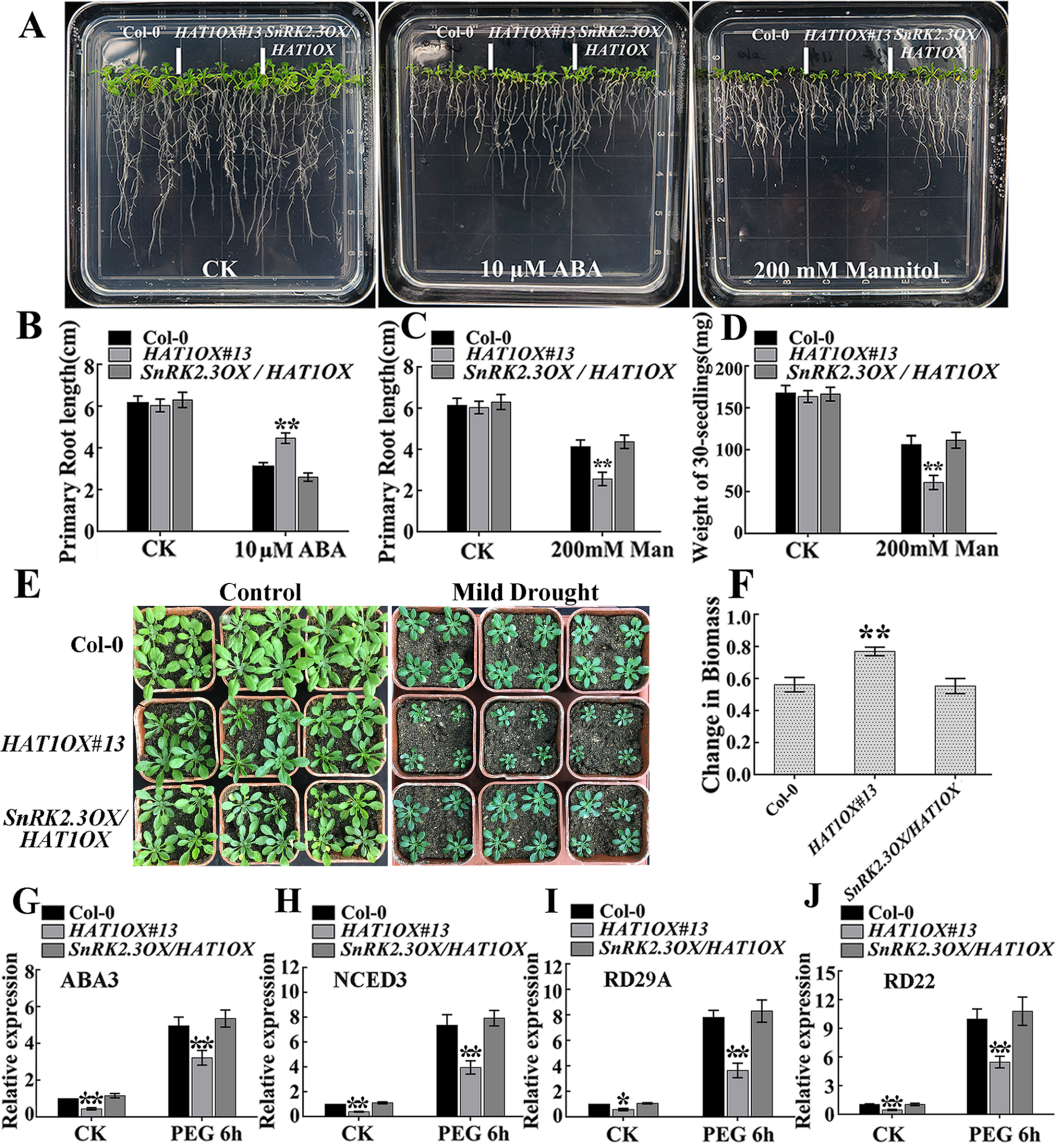
Overexpression of *SnRK2*.*3* suppresses the ABA-insensitive and drought- hypersensitive phenotypes of *HAT1*-overexpressing line. (A) Phenotypic comparison. 4-day-old Col-0, *HAT1OX#13*, and *SnRK2*.*3OX/HAT1OX* seedlings were transferred to 1/2 MS medium or 1/2 MS medium supplemented 10μM ABA or 200 mM mannitol for 10 days, and then the photos were taken. (B-D) Quantitication of primary root length and biomass in Col-0, *HAT1OX#13*, and *SnRK2*.*3OX/HAT1OX* seedlings after ABA treatment and mannitol treatment indicated in (A). Data represent mean ± SD of three independent replicates. asterisks indicate significant differences compared with Col-0 under the same treatment conditions. The significant difference was analyzed by Student’s t test (*P < 0.05, **P < 0.01). (E, F) Growth of Col-0, *HAT1OX#13*, *SnRK2*.*3OX/HAT1OX* in response to mild drought stress. 3-week-old plants were subjected to mild drought treatment and the images of both drought-treated plants (right) and the well-watered plants (left) were taken (E) and the reduction in biomass of each genotype was measured (F). The average and SDs were from three independent experiments and At least 12 plants were measured for each genotype per replication. The significance of difference was analyzed by Student’s t test (**P<0.01). (G-J) Expression of ABA or drought-responsive genes in 12-day-old seedlings with or without 15% PEG 6000 treatment for 6h. Data are means of three replicates ± SD, asterisks indicate significant differences compared with Col-0 under same conditions (Student’s t-test: * P < 0.05, **P < 0.01).

To confirm the regulation of HAT1 by SnRK2.3, we examined whether or not overexpression of *SnRK2*.*3* can suppress *HAT1OX* phenotypes in ABA and drought responses. *SnRK2*.*3OX/HAT1OX* double overexpressing line displayed an enhanced ABA sensitivity in seedlings growth and was more tolerant to drought stress compared with *HAT1OX*, which was similar to Col-0 (Fig 8A–8D and S9 Fig). Moreover, *SnRK2*.*3OX/HAT1OX* showed less reduction in biomass under mild drought conditions compared to *HAT1OX* (Fig 8E and 8F). Then, we tested the influence of SnRK2.3 overexpression on HAT1 in the regulation of ABA or drought inducible marker genes expression. As shown in Fig 8G–8J, the expression of *ABA3*, *NCED3*, *RD29A*, and, *RD22*, were significantly up-regulated in *SnRK2*.*3OX/HAT1OX*, compared to *HAT1OX*, which reached to the expression level of Col-0. These data together with phenotype tests indicated that SnRK2.3 overexpression suppressed the ABA-insensitivity and drought-hypersensitivity of *HAT1OX*.

## Discussion

Currently, the most thoroughly understood in transcriptional regulation of ABA-mediated drought responses is AREB/ ABFs pathway, which activated the expression of drought-responsive genes in an ABA-dependent manner [40], however, the components involved in compromising drought response were less well studied. In this study, we identified SnRK2.3 interaction transcription factors HAT1 and HAT3 as important components to regulate ABA-mediated drought response. As negative regulators, HAT1 and HAT3 suppressed ABA sensitivity and drought tolerance. Furthermore, we found HAT1 was a substrate of SnRK2.3 and SnRK2.3 phosphorylation decreased HAT1 protein stability and binding activity. Our results identified a new negative component that regulates ABA signaling in Arabidopsis in response to drought and established a novel mechanism to attenuate stress response.

HAT1 plays important roles in phytohormone-regulated developmental processes and stress response [23,25]. HAT1 interacts with BES1, a central regulator in BR signaling pathway, and functions as a BES1 co-repressor to inhibit BR-repressed genes and thus optimizes BR-regulated plant growth [30]. In addition, HAT1 acts as a repressor in plant defense response to CMV infection [31]. Thus, HAT1 may function as a transcriptional regulator to modulate plant growth and stress response. Several lines of evidence support the role of HAT1 as a negative regulator in ABA-mediated drought response. First, the expression of both *HAT1* and its close homologs *HAT3* is repressed by ABA and osmotic stress, indicating that these genes are ABA or stress-responsive factors. Second, HAT1 can bind to specific DNA sequences (HB binding sites) on promoter of *NCED3* and *ABA3*, two key ABA biosynthesis genes, and represses these genes expression, leading to a reduction of ABA synthesis. In addition, drought-responsive genes like *RD22* and *RD29A*, were also suppressed by HAT1. Third, consistent with the role of negative regulators for ABA signaling under stress conditions, *HAT1OX* displayed reduced sensitivity to ABA and less tolerance to drought stress, whereas the double knockout mutant *hat1hat3* showed an enhanced ABA sensitivity and increased drought tolerance phenotypes. Finally, the modulation of HAT1 by SnRK2.3 kinase further suggests that HAT1 forms part of ABA signaling network to regulate ABA-dependent stress response.

Besides the repression by ABA at transcription level, HAT1 is regulated by ABA-activated SnRK2 kinases through a post-transcriptional modification mechanism. Post-translational modifications of transcription factors fine-tune their functions to effectively and precisely implement the stress response. SnRK2s-mediated phosphorylation of target proteins triggers most of the molecular actions of ABA signaling pathway [14,41,42]. In addition to the originally identified bZIP transcription factors AREBs (ABA-Responsive Element Binding factors) that function in ABA-responsive gene regulation, 58 putative substrates of ABA-activated SnRK2s were identified through mass spectrometry-based global phosphorylation profiling, which include components involved in flowering time regulation, RNA and DNA binding, miRNA and epigenetic regulation, signal transduction, chloroplast function, and many other cellular processes [41]. In this study, we identified an additional substrate for SnRK2.3 kinase. In contrast to bZIP transcription factors AREBs, which are stabilized by SnRK2s phosphorylation [43,44], SnRK2.3 phosphorylation promotes the degradation of HAT1. In addition to destabilizing HAT1 protein, we found that SnRK2.3 phosphorylated HAT1 on its homeodomain, which is responsible for specific DNA binding, leading to the reduction of its binding ability to the HB sites on the promoter of target genes. Our results thus suggest that SnRK2.3 phosphorylation of HAT1 can have different functional consequences, inhibiting both its DNA binding and protein accumulation. However, the mechanisms how phosphorylation by SnRKs mediates HAT1 degradation remain to be determined in future studies.

HAT1 belongs to Class II HD-ZIP transcription factors, which have been shown to regulate plant growth and development [45–47]. For example, ATHB4, ATHB2 and HAT3 are required for normal leaf development and blade growth [45]. ATHB4, a shade signaling component, acts redundantly to other members of the HD-Zip class-II subfamily to integrating shade perception and hormone-mediated growth [29]. *HAT2* is an auxin inducible gene and modulates auxin-mediated morphogenesis [48]. In addition to the regulation of plant growth and development, several of the class II HD-ZIP transcription factors have been also reported to participate in plant responding to exogenous ABA and drought stress. ATHB17 has been characterized as a positive regulator of ABA response and multiple stress responses [46,49]. *ABIG1/HAT22* is induced by ABA and drought stress, and relays ABA signaled growth inhibition and drought induced senescence [50]. HDG11 can promote main root elongation and lateral root formation in *Arabidopsis* and was able to confer drought tolerance in Arabidopsis, tobacco, rice, sweet potato, cotton and woody plant poplar (Populus tomentosa Carr.) [51–55]. It seems likely that a general role for HD-ZIP II proteins is to link environmental and developmental signals to growth control. As noted above, these class II HD-ZIP transcription factors share many similar characteristics though they have different expression patterns. Expression pattern of HAT1 and HAT3 in response to BR and ABA is analogous and functions in BR-mediated hypocotyl elongation and ABA-induced drought stress tolerance are redundant. So it proposed that HAT1 together with HAT3 played essential roles in balancing plant growth and stress responses. However whether ABA regulates HAT1 and HAT3 function and stability in a similar manner is unclear and further study will be needed.

**Fig 9:**
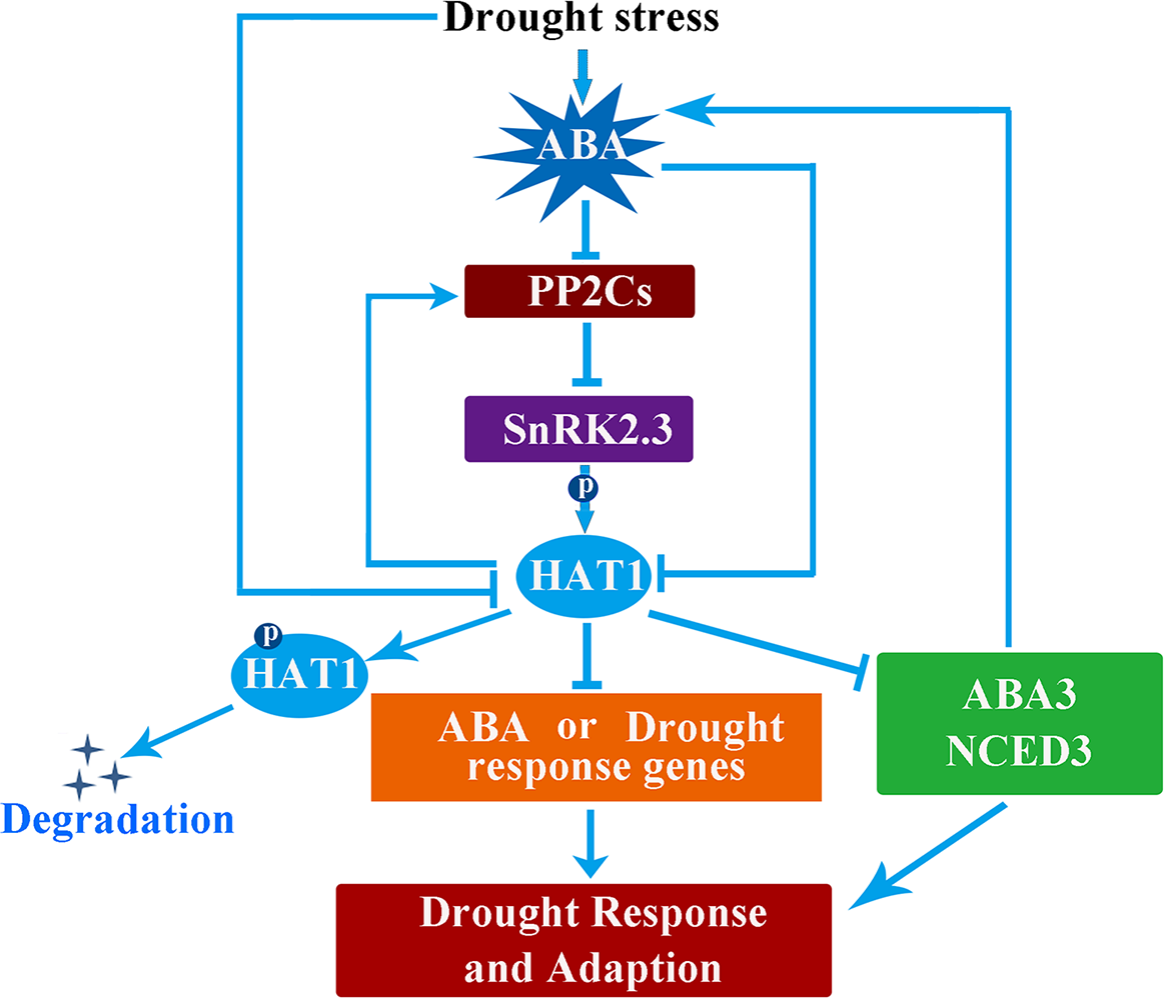
Working model for HAT1 in the negative regulation of stress responses. ABA, which is induced by drought stress, inhibits the activity of PP2Cs to release the kinase activity of SnRK2s kinase for further activation of downstream regulators through phosphorylation. HAT1 could directly bind to the promoters of *ABA3* and *NCED3* and negatively regulates the expression of these two genes, resulting in reduced ABA biosynthesis. Furthermore, HAT1 could positively regulate several *PP2C* genes expression, which in turn negatively regulates the ABA signaling. Thus, HAT1 acts as a repressor to keep the drought response silence during the environmental conditions are comfortable. Under stress conditions, stress-induced ABA accumulation could repress HAT1 at transcriptional and protein level, ensuring plants survive stress conditions. P, Phosphorylation.

Our results strongly indicate that HAT1 is an important part of mechanisms that functions to control basal ABA signaling and drought response. HAT1 can suppress ABA synthesis and signaling through down-regulating the expression of *ABA3* and *NCED3* via directly binding to their promoters, and ABA/drought-responsive genes, *RD29A* and *RD22*. In contrast, HAT1 promotes the expression of *PP2Cs* which negatively regulate the ABA response, enhancing the negative regulation of ABA signaling (Fig 9). When exposed to drought conditions, stress-induced ABA led to activation of SnRK2s, which in turn negatively regulates HAT1 functions by posttranslational regulation of its stability and binding ability. The suppression of HAT1 at both transcriptional and protein level appears to be an adaptive strategy of plant responses to water deficit, facilitating plants survival under drought conditions (Fig 9). When the environmental conditions are favorable, HAT1 and its homologous function to suppress drought response, prevent unnecessary activation of stress response, and ensure the normal growth of plants. HAT1 thus can be considered as a brake to fine tune ABA signaling and drought response (Fig 9).

In summary, this study revealed the mechanism of the negative regulatory function of HAT1 in ABA-mediated drought response (Fig 9). We found that ABA biosynthesis and signaling were repressed by HAT1. We also establish that HAT1 is phosphorylated by SnRK2.3 kinase and that SnRK2.3 phosphorylation promotes the proteasome-mediated HAT1 degradation and represses the binding ability of HAT1. The identification of negative regulators, like HAT1, and elucidation of the regulatory mechanism will lead to a better understanding of ABA signaling mechanism and drought response, which has potential in manipulating crop plants for drought tolerance.

## Materials and methods

### Plant materials and growth conditions

Arabidopsis thaliana ecotype Columbia-0 (Col-0) was used as the WT control. The *HAT1*-overexpressing lines (*HAT1-OX#11* and *HAT1-OX#13*) were described previously [30]. T-DNA insertion mutants *hat1*, *hat2* and *hat3* were obtained from ABRC (Arabidopsis Biological Resource Center) [56], corresponding to line SALK_059835, SALK _091887 and SALK_056541. We performed cross to create the double mutant *hat1hat2*, *hat1hat3* and triple mutant *hat1hat2hat3*. *HAT1OX* and mutants were identified (S2 Fig). All the plants were grown on half-strength MS plates and/or in soil under long-day conditions (16 h light/8 h dark) at 22°C.

### Construction of plasmids and generation of transgenic plants

Gene-specific primers HAT1 were used to isolate HAT1, from a cDNA library by PCR. To generate the pZP211-HAT1-GFP, full-length HAT1 was amplified and cloned into the pZP211 vector with a GFP tag using the BamHI and SalI sites [57]. To generate the Myc-SnRK2.2/2.3/2.6, the coding regions of SnRK2.2/2.3/2.6 were cloned into pCAMBIA1307-63 Myc vector [58]. To generate HAT1-Promoter::GUS, 1.3-kb fragments upstream of HAT1 were amplified by PCR using primers HAT1p-F/R and inserted into the binary vector pBI121-GUS using HindIII and BamHI sites [59]. For BiFC assays, SnRK2.2/2.3/2.6 were cloned into the pXY103 vector fused to the C terminus of YFP, and HAT1 and its fragments were fused into the pXY104 vector fused to the N terminus of YFP [60]. For the recombinant protein and GST pull-down assay, the HAT1 coding region was amplified from Col-0 cDNA and various deletion constructs were incorporated into the pETMALc-H vector (MBP, BamHI/SalI) [61]. The coding regions of SnRK2s were inserted into the binary vector PGEX-6P-1(GST, BamHI/SalI). All primers are listed in S1 Table. The construct of HAT1-GFP driven by 35S promoter were transformed into *Agrobacterium tumefaciens* (strain GV3101), which were used to transform plants by the floral dip method. Transgenic lines were selected on half-strength MS medium that contained 50 μg ml^-1^ kanamycin. Transgene expression was analyzed by western blotting.

### GUS staining and transient expression in protoplasts

Rosette leaves of 4-week-old *A*. *thaliana* plants grown under short day conditions were used for the isolation of protoplasts [62]. The relevant vectors 35S:HAT1–GFP, and 35S:GFP were used for protoplast transformation. A fluorescence microscope was used to observe GFP signals (Kim et al., 2001; Bae et al., 2008). For GUS staining, the transgenic plants with or without ABA and osmotic stress treatment were immersed in a staining solution (100 mM sodium-phosphate buffer, pH 7, 1 mM K_4_Fe(CN)_6_, 1 mM K_3_Fe(CN)_6_, 0.1% Triton X-100, 2 mM X-Gluc) overnight at 37°C in the dark followed by two times washes with 70% ethanol to remove chlorophyll. Samples were photographed using a stereoscope (Leica) equipped with a CCD camera. To test for GUS expression before and after ABA and osmotic stress, plants were treated with 100 μM ABA for 3 h and mannitol treatment for 6 h, respectively.

### Phenotype analysis and drought stress treatment

For ABA sensitivity, different genotype seeds were grown vertically on 1/2 MS medium for 3–5 days and then transplanted to normal 1/2 MS medium or 1/2 MS medium containing 10μM ABA. The root growth was observed after about 10 days [63]. For the osmotic stress treatment, 4-day-old seedlings grown on half-strength MS medium (0.5% agar) were transferred to new agar plates containing 200 mM mannitol, and the primary root length and 30-seedlings fresh weight were measured after 10 days. The primary root lengths were measured with ImageJ (National Institutes of Health, Bethesda, MD, USA). Three independent experiments were performed.

To study the promotion of stomatal closure by ABA, fully expanded young leaves of 4-week-old Arabidopsis plants were harvested and incubated in MES-KCl buffer (50 mM KCl, 10 mM MES-KOH, pH 6.15), at 22°C and exposed to light for 2 h. Once the stomata were fully open, leaves were incubated in MES-KCl buffer alone or containing 50 μM ABA. Control treatments involved the addition of DMSO, an appropriate solvent with ABA. After treatment for 3h under light conditions, the epidermal strips were immediately peeled carefully from the abaxial surface of leaves, and stomatal apertures were measured with an optical microscope (Nikon, Optiphot-2) fitted with a camera lucida and a digitizing table linked to a personal computer [64]. The stomatal aperture sizes were analysed by the software image J. To avoid any potential rhythmic effects on stomatal aperture, experiments were always started at the same time of the day. Blinded stomatal aperture experiments were conducted by another group in the laboratory who are not aware of any information about the control group (WT) and test group (mutants and transgenic plants) (S2 Data Blinded experiments). For the ROS accumulation assay in guard cells, prepared epidermal peels with or without ABA treatment were loaded with 50 μM 2,7-dichlorofluorescin diacetate for 10 min (H2DCF-DA; Sigma-Aldrich) in dark, as described previously [65]. Fluorescence emission of guard cells was analyzed using image J. Three independent experiments were performed.

To measure leaf water loss, rosette leaves of similar developmental stages from 4-week-old plants were excised from their roots, placed in open Petri dishes, and kept on the lab bench for the indicated time, and then their fresh weights were monitored, with three replicates per time-point [66]. Water loss was expressed as a percentage of weight loss at the indicated time versus initial fresh weight.

For the progressive drought treatment experiment, 10-day-old plants were transferred from 1/2 MS medium to water-saturated soil and the plants were grown in the same glasshouse with 120 μmol m^-2^ s^-1^ under a 16 h: 8 h, light: dark photoperiod (23°C) for 2 weeks, then the plants were deprived of water for 14 days and the survival rates of plants were determined 5 d after re-watering (rehydration) [67]. Relative electrolyte leakage rates were measured as described by Julieta V. Cabello et al. [68]. Three independent experiments were performed.

The mild drought treatment was conducted as previously described [69,70], with a slight modification. Briefly, 12-day-old Arabidopsis seedlings of different genotypes grown on 1/2 MS medium were transferred to pots. Before transfer, the relative water content of the pots was set at 2.2 g water g^-1^ dry soil. The plants were kept to grow for 10 days. During this growth period, the water content of the soil was kept constant until 10 days, after which it was lowered daily to target 0.7 g water g^-1^ dry soil and mild drought stress treatment began. Control soil water content (well water) was maintained at a constant value of 2.2 g water g^-1^ dry soil during the entire experiment. Fig 3D showed the water loss from the peat pellets during the duration of the experiment. After mild drought treatment for 9 days, images of each genotype were taken. To quantify the biomass change of each genotype, the dry weights of detached rosettes of both the drought-treated and the well-watered control were measured. The reduction in biomass was calculated using the following equation:

Reduction in Biomass (RB) = (Biomass of Well Watered Control–Biomass of Drought Treated) / (Biomass of Well Watered Control)

### PEG treatment and ABA content determination

Polyethylene glycol (PEG) 6000 was used to mimic drought stress [66]. Arabidopsis seedlings grown on 1/2 MS medium plates were transferred to 1/2 MS liquid medium (CK) and 1/2MS liquid medium containing 15% PEG (drought stress treatment) for indicated time, and then the seedlings were harvested for gene expression analysis or ABA content assay.

For ABA content assay, 0.5g 12-day-old seedlings with or without 15% PEG treatment were homogenized in 2 mL of 80% methanol, and incubated with additional 3 mL of 80% methanol overnight at 4°C. After centrifugation (4000 r/min for 10 min, 4°C), the supernatant was passed through a C18-SepPak classic cartridge (Waters, Milford, USA) [71]. ABA content measurement was performed by using a Plant hormone abscisic acid (ABA) ELISA Kit (BIOSAMITE, CK-E90047). Three independent experiments with different biological repeats were done.

### RNA extraction and reverse-transcription PCR

12-day-old seedlings grown under long-day conditions were used for qRT-PCR analysis of ABA or drought stress-responsive genes. Total RNA extraction, cDNA synthesis and qRT-PCR were performed as described by Zhang et al. (2010) [72]. Briefly, total RNAs were extracted using RNAprep pure Plant Kit (from Transgene Biotech Co. Ltd. of Qiagen, Beijing) according to the manufactures’ protocols. Total RNAs treated with DNase I (Transgene Biotech Co. Ltd. of Qiagen, Beijing) were converted into cDNAs using M-MLV Reverse Transcriptase Kit (Invitrogen, USA). Real-time qPCR analysis was carried out using the SYBR® Premix Ex TaqTM II (TAKARA) on a BIO-RAD CFX Connect^TM^ Real-Time System, following the manufacturer’s instruction. Three independent experiments were performed, and three technical replicates of each experiment were performed. *Actin2* genes was used as an internal control for normalization of transcript levels [73]. All primers used for gene expression analysis are shown in S1 Table.

### Protein interaction assay

For GST pull-down assay, HAT1 and HAT1 fragments fused with MBP were purified with amylose resin (NEB). SnRK2.3 fused with GST was purified with glutathione beads (Sigma, G4510). GST pull-down assays were performed as described Yin et al. [74]. The assays were repeated three times with similar results.

For the BiFC assay, SnRK2s were cloned into the pXY103 vector and fused to the C terminus of YFP, and HAT1 and its fragments were fused into the pXY104 vector and fused to the N terminus of YFP. The resulting plasmids were introduced into Agrobacterium tumefaciens (strain GV3101), and then infiltrated into young leaves of *Nicotiana benthamiana*. Infected leaves were analyzed 48h after infiltration. YFP fluorescence was observed under a fluorescence microscope (Leica).

For the Co-IP assays in the Arabidopsis protoplasts, full-length coding sequences of HAT1 and SnRK2.3 were individually cloned into tagging plasmids behind Flag or Myc tag sequences in the sense orientation behind the cauliflower mosaic virus 35S promoter. Flag-fused HAT1 and Myc-fused SnRK2s were then transformed into Arabidopsis protoplasts. After overnight incubation at 23°C, the protoplasts were lysed, sonicated, and centrifuged. Co-IP assays were performed using transiently expressed proteins as described previously [75]. Briefly, the protein extracts were mixed with Myc agarose beads (Sigma-Aldrich) and then incubated at 4°C for 2 h. After being was hed at least five times, the agarose beads were recovered and mixed with the SDS sample buffer. The samples were detected by immunoblotusing anti-Myc antibody, and the coimmunoprecipitated protein was then detected using an anti-Flag antibody.

### In vitro kinase assay and detection of in vivo HAT1 phosphorylation

The in vitro kinase assay was performed as previously described as Yin et al. [74]. MBP, MBP-HAT1, and truncated MBP-HAT1 were incubated with GST-SnRK2.3 kinase in 20 μL of kinase buffer [20 mM Tris (pH 7.5), 100 mM NaCl, and 12 mM MgCl_2_] and 10 μCi ^32^P ATP. After incubation at 37°C for 60 min, the reactions were stopped by adding 20 μL of 2×sodium dodecyl sulfate (SDS) buffer and boiling for 5 min. Proteins were resolved by polyacrylamide gel electrophoresis (PAGE) and phosphorylation was detected by exposing to a phosphor screen, and signals were obtained by a Typhoon 9410 phosphor imager. The in vivo phosphorylated HAT1 was examined by Phostag reagent (NARD Institute) with or without CIP treatment as described Guan et al [76].

### Protein extraction and immunoblot analysis

Total protein was extracted from Arabidopsis using extraction buffer as described previously [77]. Briefly, plant material was ground in the Eppendorf tube using 2×sodium dodecyl sulfate (SDS) sample buffer, centrifuged at 13,000g for 10 min, and the supernatant was saved. For immunoblot analysis, total protein was separated by 10% SDS-polyacrylamide gel electrophoresis (PAGE) and transferred to PVDF membranes. The membrane was blocked for 1 h in TBST buffer (10 mM Tris, pH 7.6, 150 mM NaCl, 1.0% Tween20) with 5% skim milk powder at room temperature and then incubated with specific primary antibodies in TBST buffer for 1 h. After the membrane washed by TBST buffer for several times, the blot was incubated with horseradish peroxide-conjugated secondary antibody (goat anti-rabbit IgG, Thermo fisher) at a dilution of 1/10000 for detection by the enhanced chemilumine scence assay.

### The EMSA and ChIP assays

EMSA was performed using an Electrophoretic Mobility-Shift Assay (EMSA) Kit *with SYBR Green and SYPRO Ruby EMSA stains* (Molcularprobes^TM^, E33075). The binding reactions were carried out in 20 μL binding buffer [25 mM HEPES-KOH pH 8.0, 50 mM KCl, 1 mM dithiothreitol (DTT) and 10% glycerol] with approximately 1 ng probe (10000 cpm) and recombinant proteins purified from E. coli. After 30 min incubation on ice, the reactions were resolved by 5% native polyacrylamide gels with 1×TGE buffer (6.6 g L^-1^Tris, 28.6 g L^-1^ glycine, 0.78 g L^-1^EDTA, pH 8.7). The assays were repeated three times with similar results.

ChIP was performed as previously described [78]. Briefly, 14-day-old seedlings of *HAT1OX* and *SnRK2*.*3OX/HAT1OX* seedlings were treated as above described. 1.5 g of the samples were cross-linked with formaldehyde and nuclei were isolated using sucrose gradients. Chromatin was sonicated to generate fragments with the average size of 300 bp and precipitated using anti-GFP antibody. Immunocomplexes were harvested by protein A beads, washed and reverse cross-linked by boiling in the presence of Chelex resin (Bio-Rad, http://www.bio-rad.com/). The level of precipitated DNA fragments was quantified by RT-qPCR using specific primer sets (S1 Table). Col-0 was the negative control and the values in control plants were set to 1 after normalization against TA3 for qPCR analysis. Three biological replicates were carried out through the whole process.

## Supporting information

S1 Fig Expression pattern of HAT1 response to ABA and drought stress and subcellular localizations of HAT1. **(**A) GUS staining for expression patterns of HAT1. Transgenic plants expressing HAT1-Promoter::GUS at seedlings (left) and leaves of adult plants (right) were stained with 5-bromo-4-chloro-3-indolyl β-D-glucuronide (X-Gluc). GUS expression was examined in cotyledons, roots and guard cells before and after 100 μM abscisic acid (ABA) treatment for 3 h and mannitol treatment for 6 h. (B) Subcellular localizations of HAT1-GFP. Protoplasts from wild-type (WT) plants were transformed with 35S:HAT1-GFP or 35S:GFP. The signals were observed under a fluorescence microscope. GFP, green fluorescent protein. Cell images were also taken under bright field as a control. Bars, 20 μm.
(TIF)

S2 Fig Indentification of T-DNA insertion mutants and *HAT1OX* lines. (A) Reverse transcription-PCR was employed to estimate the transcription levels of *HAT1*, *HAT2* and *HAT3* in T-DNA insertion mutants. (B) HAT1 protein was detected by western blotting with anti-GFP antibody. Similar HAT1 protein levels in *HAT1OX#11* and *HAT1#13* line.
(TIF)

S3 Fig The ABA sensitivity and osmotic tolerance of HAT1 and its homologous genes T-DNA insertion mutants (*hat1, hat2, hat3, hat1hat2, hat1hat3, hat1hat2hat3*). (A, B) Expression patterns of *HAT2* and *HAT3* in response to ABA and osmotic stress. 12-day-old Col-0 seedlings were transferred to liquid MS medium containing 100 μM ABA and 200 mM mannitol and then the plants were harvested at the indicated time. *Actin2* was used as the internal control. Data are shown as mean ± SD of three independent experiments. (C) Growth of different genotype seedlings on 1/2 MS medium with/without 10μM ABA or 200 mM mannitol. The 4-day-old seedlings were transferred to 1/2 MS or 1/2 MS medium supplemented 10μM ABA or 200 mM mannitol for 10 days, and then the photos were taken. (D-F) Quantification of primary root length and biomass in different genotypes after ABA treatment or mannitol treatment indicated in (C). The average and SDs were from three replications, asterisks indicate significant differences compared with Col-0 under the same treatment conditions. The significant difference was analyzed by Student’s t test (*P < 0.05, **P < 0.01).
(TIF)

S4 Fig The role of *HAT1* and its homologous genes (*HAT2*, *HAT3*) in abscisic acid (ABA)-induced stomatal closure. (A) Epidermal peels of indicated genotypes were treated with or without ABA for 2 h after stomatal pre-opening under light for 3h, and the stomatal aperture was measured by microscope. Scale bars: 10μm. (B) Stomatal apertures of different genotypes indicated in (A). Bars indicate SD calculated from three replications and at least 20 stomatals were measured for each genotype per replication. The significance of difference was analyzed by Student’s t test (**P < 0.01).
(TIF)

S5 Fig Drought tolerance of Col-0, *HAT1OX* lines and *hat1* mutant plants (*hat1, hat1hat3*). (A) Phenotypes of different genotypes in response to progressive drought stress. 4-week-old plants were subjected to drought stress by withholding watering (drought) for 14 days (when the lethal effect was observed in the Col-0), followed by rehydration for 5days. (B) Membrane stability status of different genotypes subjected to drought stress for 7 days and 14 days. Data is presented as a percentage of electrolyte leakage. Data are shown as mean SD of three independent experiments. The significance of difference was analyzed by Student’s t test (*P < 0.05, **P < 0.01). (C) Percentage of plants that survived the treatment mentioned in (A). Survival rate was recorded 5 days after rewatering. Bars indicate SD calculated from three replicated experiments. The significance of difference was analyzed by Student’s t test (*P < 0.05, **P < 0.01).
(TIF)

S6 Fig HAT1 interacts with SnRK2.3 in its N-terminal region. (A) Schematic representation of a series of truncation mutations of HAT1. HD, homeodomain; LZ, leucine zipper domain. (B) BiFc assay for the interaction of SnRK2.3 with HAT1 fragments. The truncated HAT1 fragments were fused with n-YFP and co-expressed with SnRK2.3-cYFP, respectively. (C) SnRK2.3 interacts with N-terminal region of HAT1 in GST pull-down assay. GST, GST-SnRK2.3 and MBP-tagged different domains of HAT1 were used in this assay. MBP-tagged domains of HAT1 were detected by western blotting with anti-MBP antibody.
(TIF)

S7 Fig SnRK2.3 interacts with HAT3 and ABA treatment promotes the degradation of HAT3. (A) BiFC analysis of SnRK2.3 and HAT3 interactions with fusions to N- and C-terminal fragments of YFP, respectively. The constructs were expressed in tobacco leaves and the reconstitution of YFP is determined. Scale bars: 50 μm. (B) Time microscope images of *Nicotianabenthamiana* leaf epidermal cells expressing HAT3-GFP exposed to 50 μM ABA. The experiment was repeated three times with similar results and representative photos were displayed. Scale bar: 50 μm.
(TIF)

S8 Fig The expression level of *SnRK2.3* and *HAT1* in *SnRK2.3OX/HAT1OX* double overexpressing plants. The expression of *HAT1* (A) and *SnRK2*.*3* (B) was tested by qRT-PCR in Col-0, *HAT1OX#13* and *SnRK2*.*3OX/HAT1OX*. Data are shown as mean ± SD and Three independent experiments were done (Student’s t-test: ** P<0.01).
(TIF)

S9 Fig Drought sensitivity of Col-0, *HAT1OX* lines SnRK2.3OX/HAT1OX plants. (A-C) Drought phenotypes (A), membrane stability status (B) and survival rates (C) of Col-0, *HAT1OX#13*, and *SnRK2*.*3OX/HAT1OX* plants subjected to progressive drought stress. In F, data is presented as a percentage of electrolyte leakage. The average and SDs were from three biological repeats in A. The significance of difference was analyzed by Student’s t test (*P < 0.05, **P < 0.01).
(TIF)

S1 Data Underlying data for Figs 1A, 1B, 1D, 1F, 2B, 2D, 2F, 2G, 3B, 3D, 3E, 4, 5B, 7D, 7E, 8B, 8C, 8D and 8F. (XLSX)

S2 Data Underlying data for S3D, S3E, S3F, S4, S5B, S5C, S8, S9B, S9C, Blinded experiments. (XLSX)

S1 Table Primer sequences used in this study. (DOCX)
