# Supplementary figures and images for: Transcription factor HAT1 is a substrate of SnRK2.3 kinase and negatively regulates ABA synthesis and signaling in Arabidopsis responding to drought
Source: PLoS Genet. 2018 Apr 16;14(4):e1007336. doi: 10.1371/journal.pgen.1007336 (PMC5919683; doi:10.1371/journal.pgen.1007336)

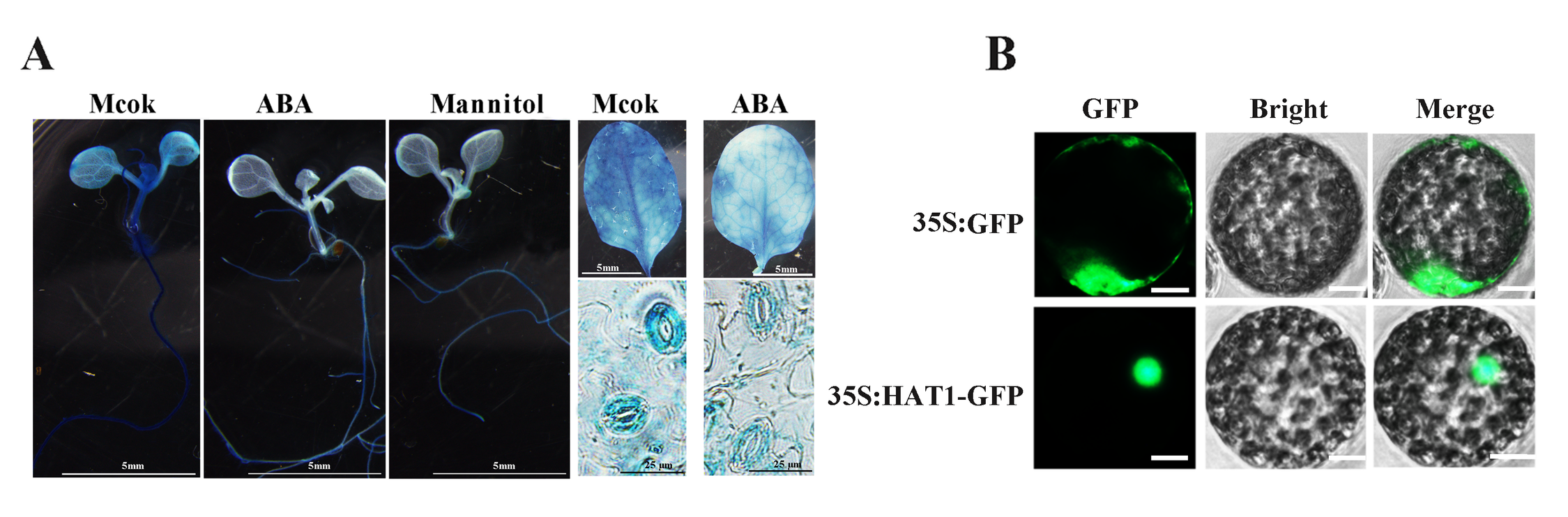

Supplement: S1 Fig — (A) GUS staining for expression patterns of HAT1. Transgenic plants expressing HAT1-Promoter::GUS at seedlings (left) and leaves of adult plants (right) were stained with 5-bromo-4-chloro-3-indolyl β-D-glucuronide (X-Gluc). GUS expression was examined in cotyledons, roots and guard cells before and after 100 μM abscisic acid (ABA) treatment for 3 h and mannitol treatment for 6 h. (B) Subcellular localizations of HAT1-GFP. Protoplasts from wild-type (WT) plants were transformed with 35S:HAT1-GFP or 35S:GFP. The signals were observed under a fluorescence microscope. GFP, green fluorescent protein. Cell images were also taken under bright field as a control. Bars, 20 μm. (TIF) [file pgen.1007336.s001.tif]

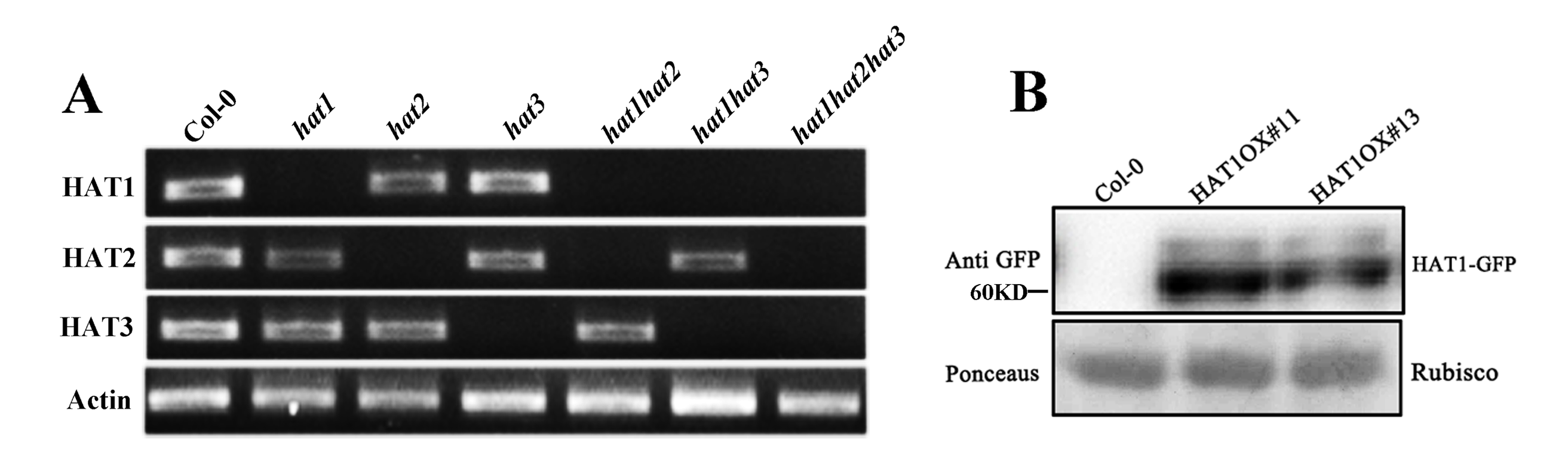

Supplement: S2 Fig — (A) Reverse transcription-PCR was employed to estimate the transcription levels of HAT1, HAT2 and HAT3 in T-DNA insertion mutants. (B) HAT1 protein was detected by western blotting with anti-GFP antibody. Similar HAT1 protein levels in HAT1OX#11 and HAT1#13 line. (TIF) [file pgen.1007336.s002.tif]

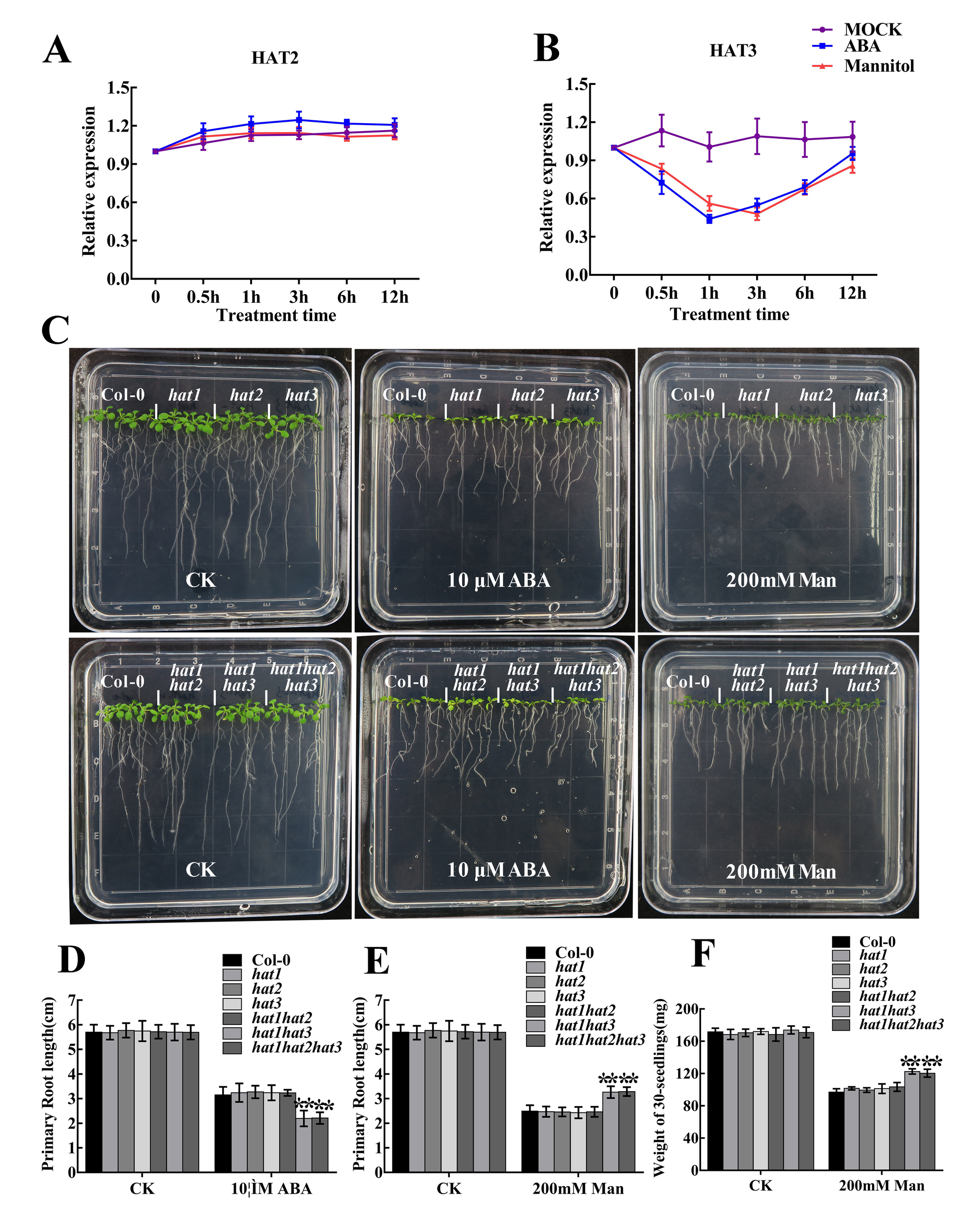

Supplement: S3 Fig — (A, B) Expression patterns of HAT2 and HAT3 in response to ABA and osmotic stress. 12-day-old Col-0 seedlings were transferred to liquid MS medium containing 100 μM ABA and 200 mM mannitol and then the plants were harvested at the indicated time. Actin2 was used as the internal control. Data are shown as mean ± SD of three independent experiments. (C) Growth of different genotype seedlings on 1/2 MS medium with/without 10μM ABA or 200 mM mannitol. The 4-day-old seedlings were transferred to 1/2 MS or 1/2 MS medium supplemented 10μM ABA or 200 mM mannitol for 10 days, and then the photos were taken. (D-F) Quantification of primary root length and biomass in different genotypes after ABA treatment or mannitol treatment indicated in (C). The average and SDs were from three replications, asterisks indicate significant differences compared with Col-0 under the same treatment conditions. The significant difference was analyzed by Student’s t test (*P < 0.05, **P < 0.01). (TIF) [file pgen.1007336.s003.tif]

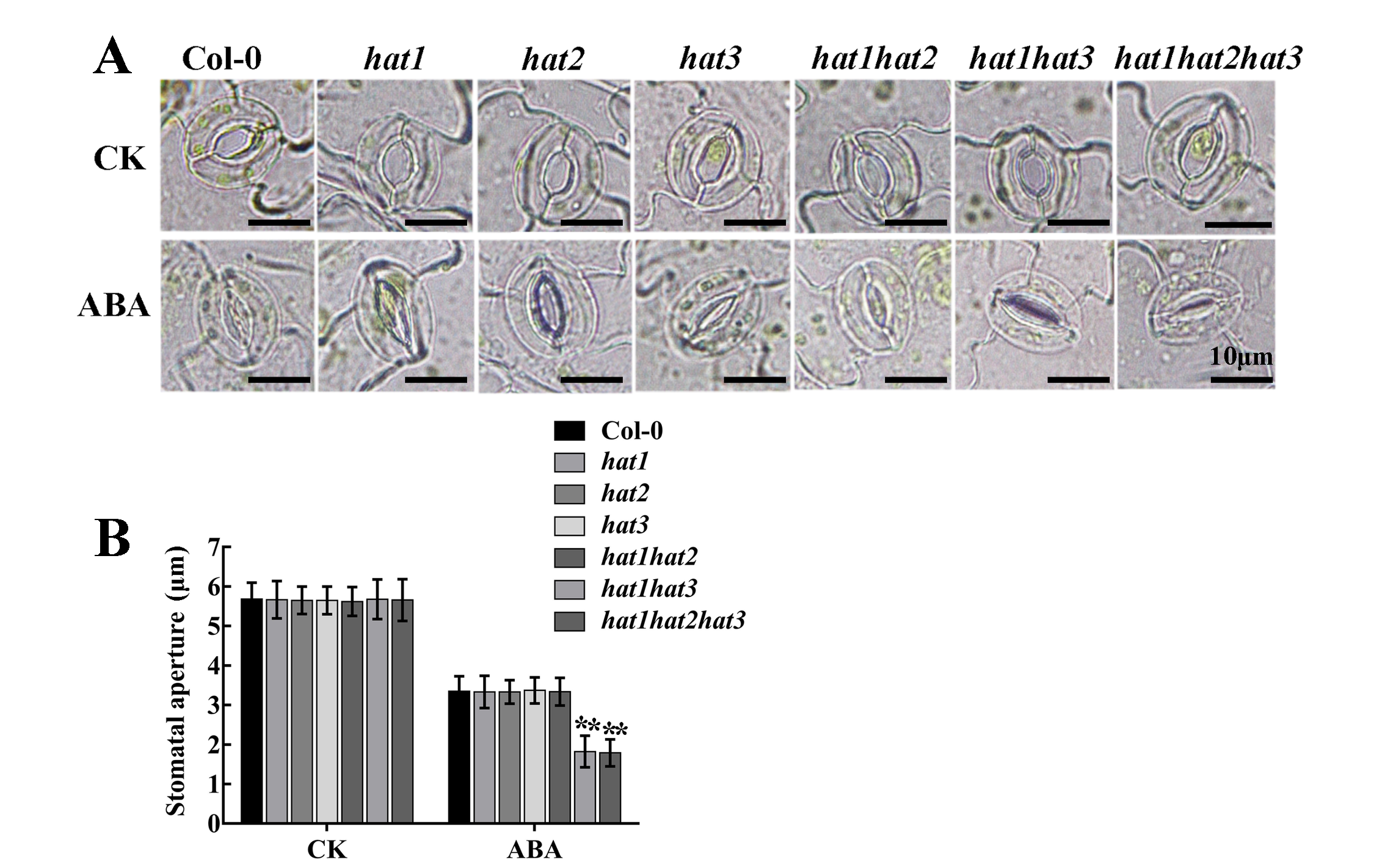

Supplement: S4 Fig — (A) Epidermal peels of indicated genotypes were treated with or without ABA for 2 h after stomatal pre-opening under light for 3h, and the stomatal aperture was measured by microscope. Scale bars: 10μm. (B) Stomatal apertures of different genotypes indicated in (A). Bars indicate SD calculated from three replications and at least 20 stomatals were measured for each genotype per replication. The significance of difference was analyzed by Student’s t test (**P < 0.01). (TIF) [file pgen.1007336.s004.tif]

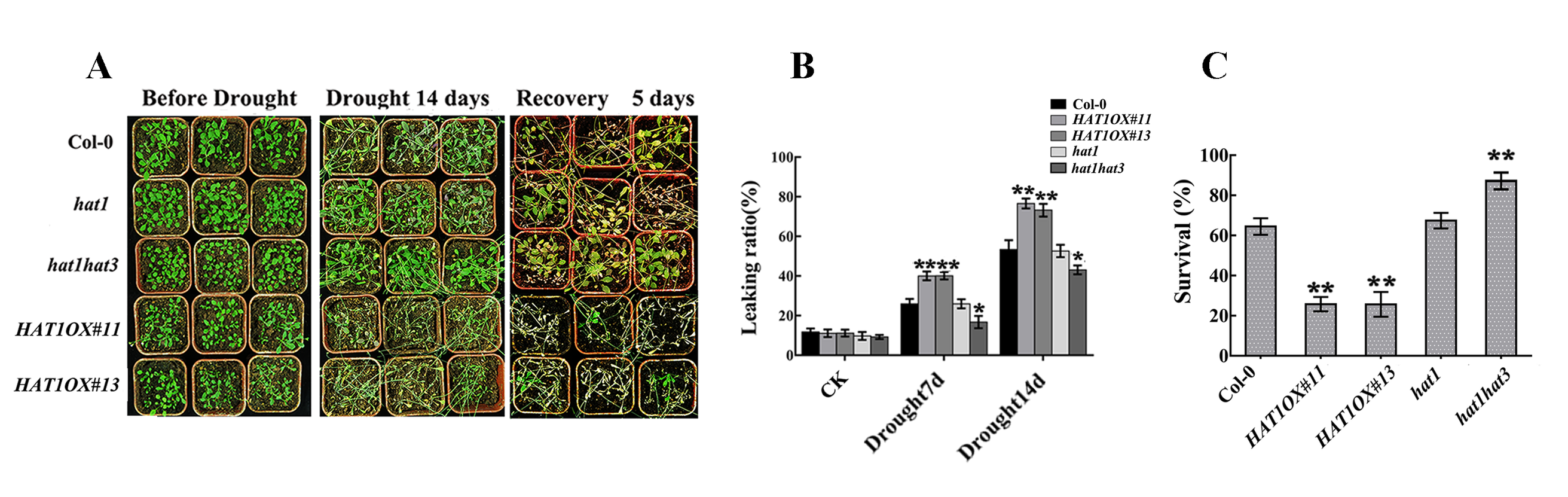

Supplement: S5 Fig — (A) Phenotypes of different genotypes in response to progressive drought stress. 4-week-old plants were subjected to drought stress by withholding watering (drought) for 14 days (when the lethal effect was observed in the Col-0), followed by rehydration for 5days. (B) Membrane stability status of different genotypes subjected to drought stress for 7 days and 14 days. Data is presented as a percentage of electrolyte leakage. Data are shown as mean SD of three independent experiments. The significance of difference was analyzed by Student’s t test (*P < 0.05, **P < 0.01). (C) Percentage of plants that survived the treatment mentioned in (A). Survival rate was recorded 5 days after rewatering. Bars indicate SD calculated from three replicated experiments. The significance of difference was analyzed by Student’s t test (*P < 0.05, **P < 0.01). (TIF) [file pgen.1007336.s005.tif]

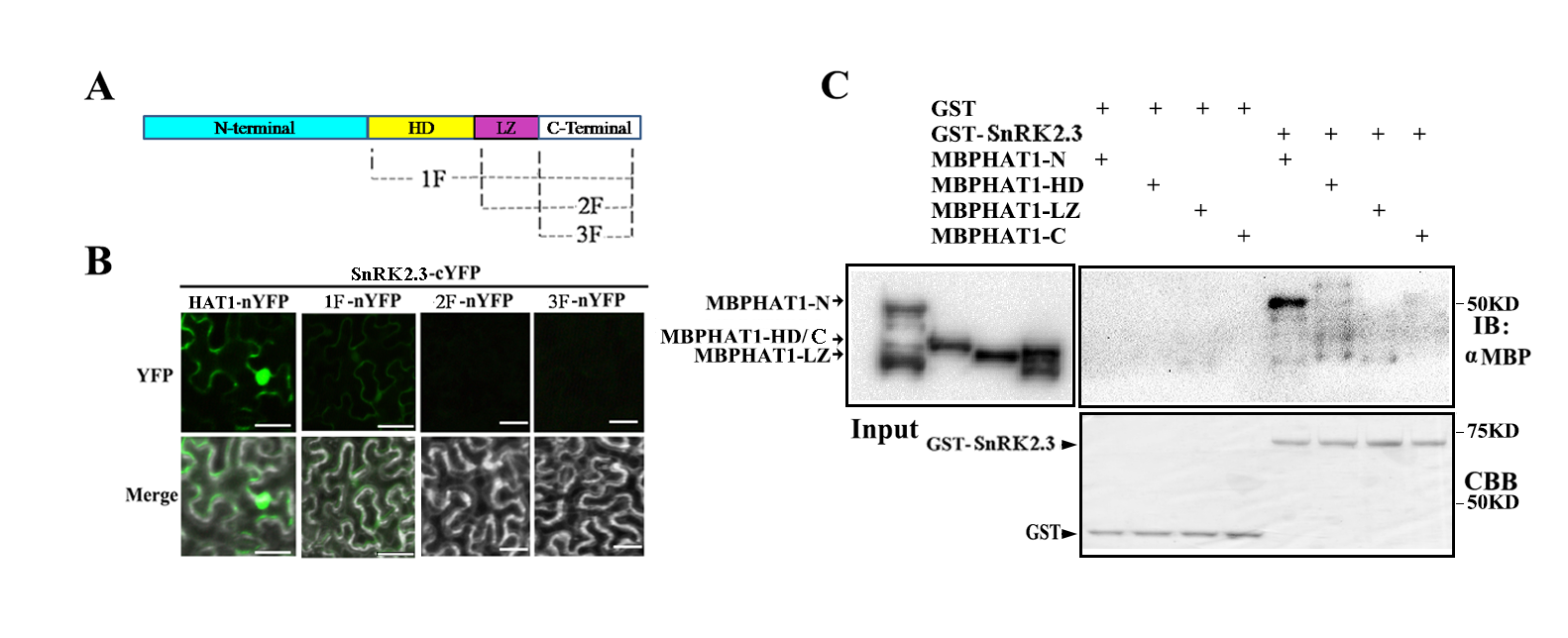

Supplement: S6 Fig — (A) Schematic representation of a series of truncation mutations of HAT1. HD, homeodomain; LZ, leucine zipper domain. (B) BiFc assay for the interaction of SnRK2.3 with HAT1 fragments. The truncated HAT1 fragments were fused with n-YFP and co-expressed with SnRK2.3-cYFP, respectively. (C) SnRK2.3 interacts with N-terminal region of HAT1 in GST pull-down assay. GST, GST-SnRK2.3 and MBP-tagged different domains of HAT1 were used in this assay. MBP-tagged domains of HAT1 were detected by western blotting with anti-MBP antibody. (TIF) [file pgen.1007336.s006.tif]

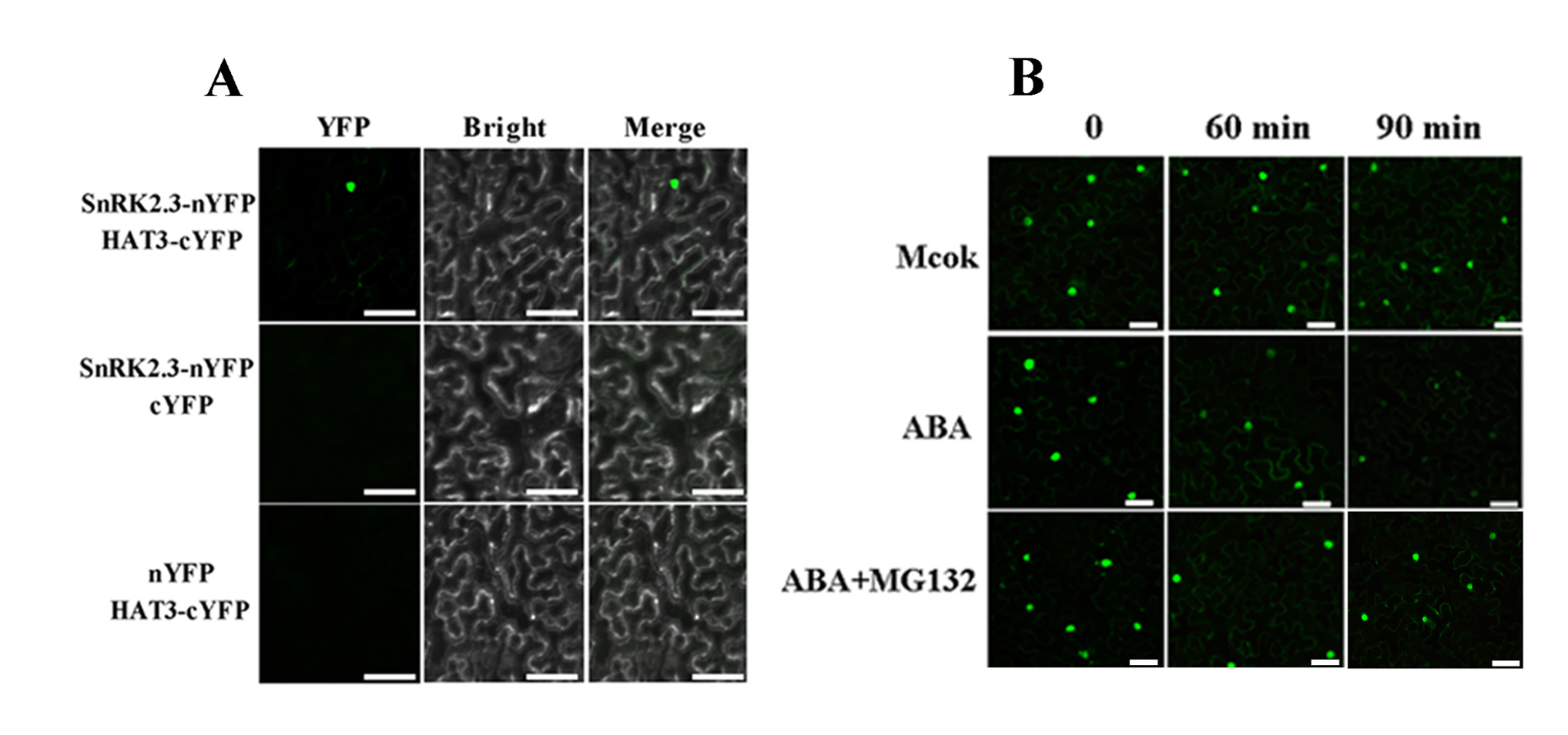

Supplement: S7 Fig — (A) BiFC analysis of SnRK2.3 and HAT3 interactions with fusions to N- and C-terminal fragments of YFP, respectively. The constructs were expressed in tobacco leaves and the reconstitution of YFP is determined. Scale bars: 50 μm. (B) Time microscope images of Nicotianabenthamiana leaf epidermal cells expressing HAT3-GFP exposed to 50 μM ABA. The experiment was repeated three times with similar results and representative photos were displayed. Scale bar: 50 μm. (TIF) [file pgen.1007336.s007.tif]

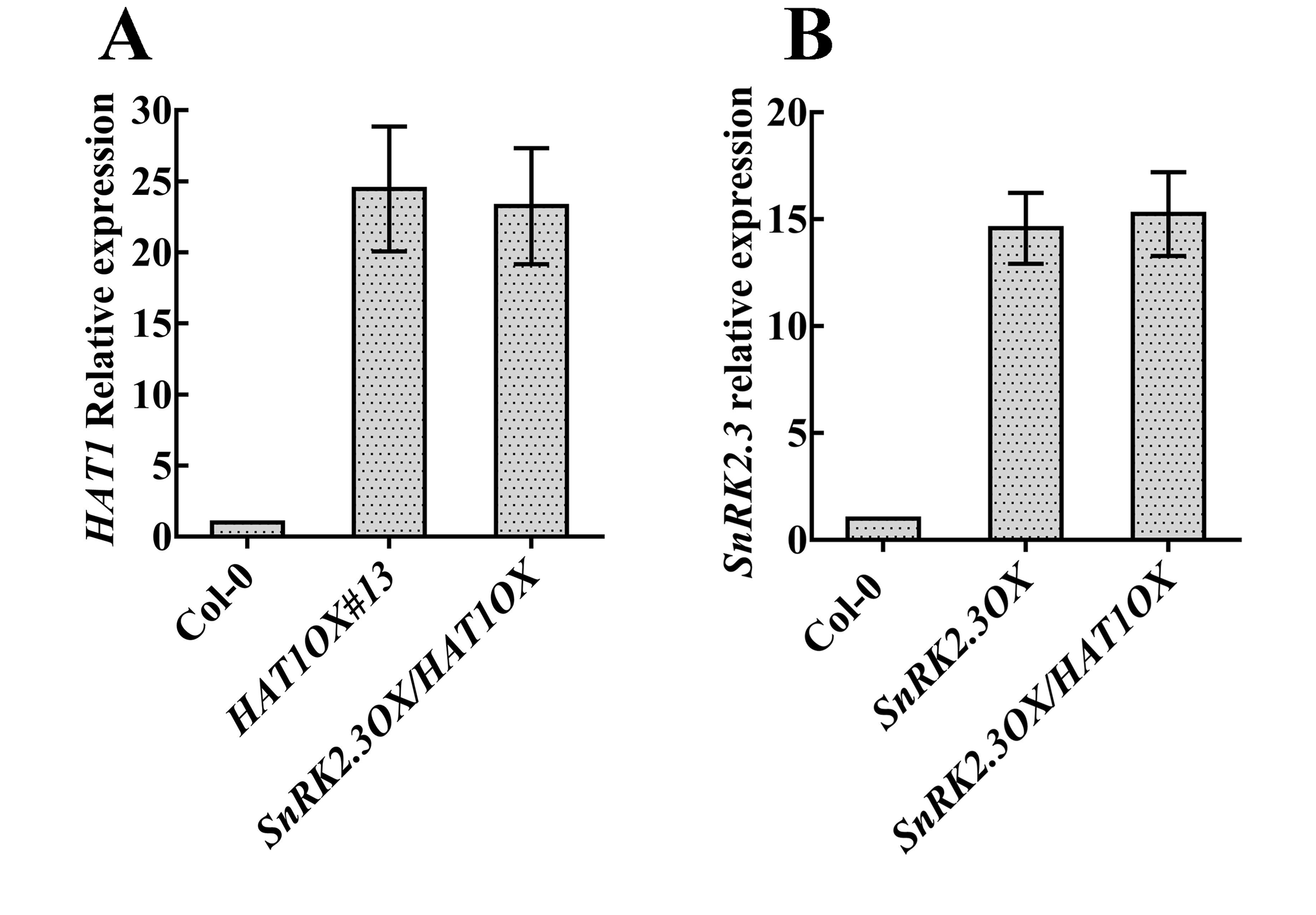

Supplement: S8 Fig — The expression of HAT1 (A) and SnRK2.3 (B) was tested by qRT-PCR in Col-0, HAT1OX#13 and SnRK2.3OX/HAT1OX. Data are shown as mean ± SD and Three independent experiments were done (Student’s t-test: ** P<0.01). (TIF) [file pgen.1007336.s008.tif]

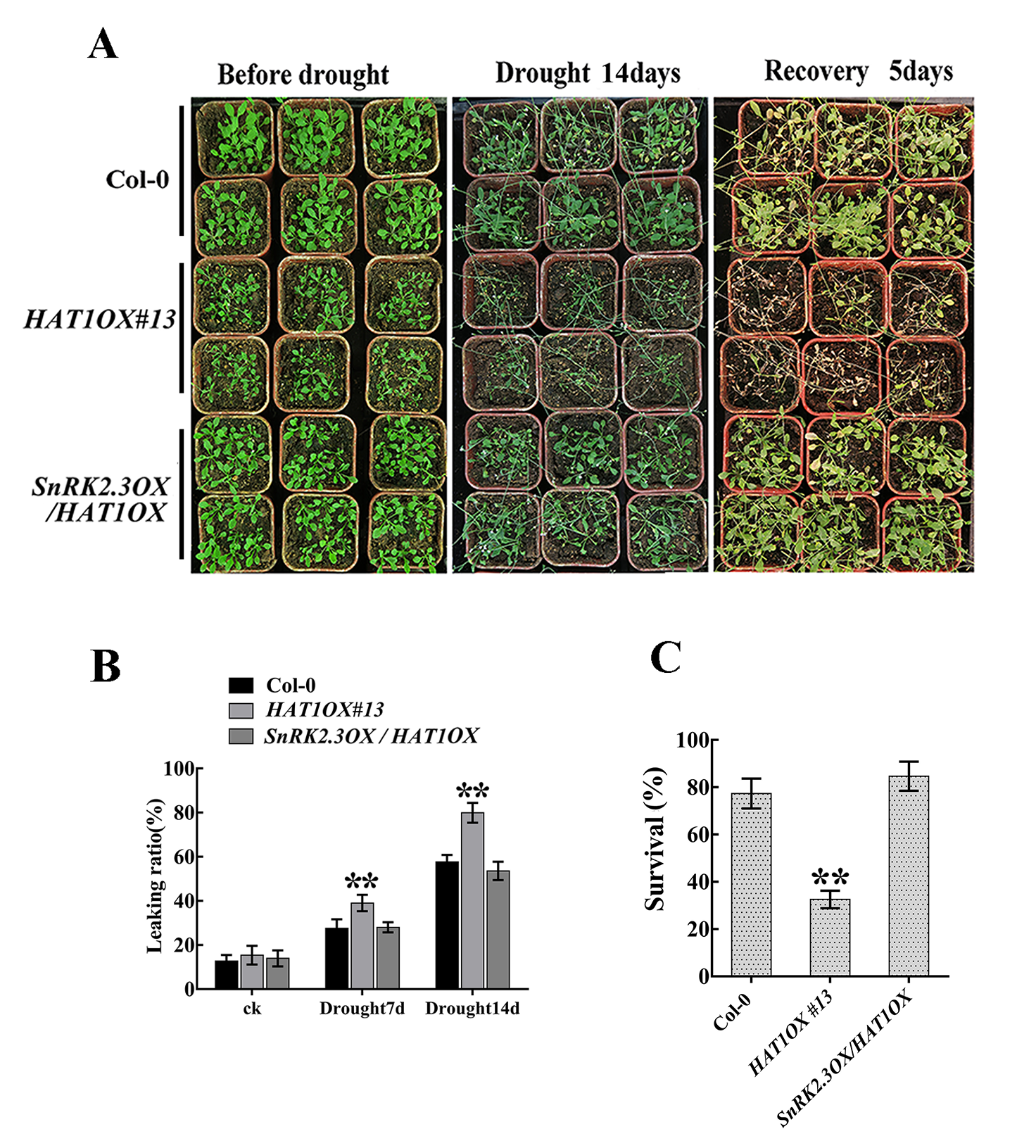

Supplement: S9 Fig — (A-C) Drought phenotypes (A), membrane stability status (B) and survival rates (C) of Col-0, HAT1OX#13, and SnRK2.3OX/HAT1OX plants subjected to progressive drought stress. In F, data is presented as a percentage of electrolyte leakage. The average and SDs were from three biological repeats in A. The significance of difference was analyzed by Student’s t test (*P < 0.05, **P < 0.01). (TIF) [file pgen.1007336.s009.tif]
