# Supplementary material for: Transcription factor HAT1 is a substrate of SnRK2.3 kinase and negatively regulates ABA synthesis and signaling in Arabidopsis responding to drought
Source: PLoS Genet. 2018 Apr 16;14(4):e1007336. doi: 10.1371/journal.pgen.1007336 (PMC5919683; doi:10.1371/journal.pgen.1007336)
Supplement: S1 Table — (DOCX) [file pgen.1007336.s012.docx]

**S1 Table. Primer sequences**

| HAT1 Overexpression | HAT1-over-BamHI-F | CGCGGATCCATGATGATGGGTAAAGAGGA |
| --- | --- | --- |
|  | HAT1-over- SalI-R | GCGGTCGACAGACCTAGGACGCATCACAT |
| HAT1-promoter:GUS | HAT1p-HindIII-F | CCCAAGCTTAATCATCTTTCGGCAGTTT |
|  | HAT1p-BamHI-R | GCGGGATCCATGAGATGTTGTCAGGTTG |
| MBP-HAT1 | MBP-HAT1-BamHI-F | CGCGGATCCATGATGATGGGTAAAGAGGA |
|  | MBP-HAT1-SalI-R | GCGGTCGACTTAAGACCTAGGACGCA |
|  | MBP-HAT1-N-SalI-R | GCGGTCGACTTAACAAGTCTCACCTCCGT |
|  | MBP-HAT1-HD-BamHI-F | GCGGGATCCAGGAAGAAGCTTAGACTAT |
|  | MBP-HAT1-HD-SalI-R | GCGGTCGACTTACTGCTTTAACTTTGTCC |
|  | MBP-HAT1-LZ-BamHI-F | GCGGGATCCACCGAAGTGGATTGCGAGT |
|  | MBP-HAT1-LZ-SalI-R | GCGGTCGACTTACATCTGACCATACAACC |
|  | MBP-HAT1-C-BamHI-F | GCGGGATCCAGTCCACCGACCACAC |
| GST-SnRKs | GST-SnRK2.2-ECORI | CCGGAATTCATGGATCCGGCGACTAATTC |
|  | GST-SnRK2.2-SalI | GCGGTCGACTCAGAGAGCATAAACTATCT |
|  | GST-SnRK2.3-BamHI-F | GCGGGATCCATGGATCGAGCTCCGGTGACC |
|  | GST-SnRK2.3-SalI-R | GCGGTCGACTTAGAGAGCGTAAACTATCT |
|  | GST-SnRK2.6-BamHI-F | CGCGGATCCATGGATCGACCAGCAGTGAG |
|  | GST-SnRK2.6-SalI-R | GCGGTCGACTCACATTGCGTACACAATCT |
| BiFc-SnRKs | BiFc-SnRK2.2-SmaI | TCCCCCGGGATGGATCCGGCGACTAATTC |
|  | BiFc-SnRK2.2-SalI | GCGGTCGACGAGAGCATAAACTATCT |
|  | BiFc-SnRK2.3-BamHI-F | GCGGGATCCATGGATCGAGCTCCGGTGACC |
|  | BiFc-SnRK2.3-SalI-R | GCGGTCGACGAGAGCGTAAACTATCT |
|  | BiFc-SnRK2.6-BamHI-F | CGCGGATCCATGGATCGACCAGCAGTGAG |
|  | BiFc-SnRK2.6-SalI-R | GCGGTCGACCATTGCGTACACAATCT |
| BiFc-HAT1 | BiFC-HAT1-BamHI-F | CGCGGATCCATGATGATGGGTAAAGAGGA |
|  | BiFC-HAT1-BamHI-1F | GCGGGATCCAGGAAGAAGCTTAGAC |
|  | BiFC-HAT1-BamHI-2F | GCGGGATCCACCGAAGTGGATTGCG |
|  | BiFC-HAT1-BamHI-3F | GCGGGATCCAGTCCACCGACCACAC |
|  | BiFC-HAT1-SalI-R | GCGGTCGACAGACCTAGGACGCATCACAT |
| Chip-qPCR | TA3ChIP-F | GATTCTTACTGTAAAGAACATGGCATTGAGAGA |
|  | TA3ChIP-R | TCCAAATTTCCTGAGGTGCTTGTAACC |
|  | ABA3-A1(Chip)-F | ACCGAACCCGACCCGAAATCT |
|  | ABA3-A1(Chip)-R | GAAATAACTTAGCTTATAGGA |
|  | ABA3-A2(Chip)-F | TTGAGGAGACAATTGACCAAC |
|  | ABA3-A2(Chip)-R | TGTATTACAAAATTGAATGGT |
|  | NCED3-N1(Chip)-F | CGAAGTATTAGAGTTCTT |
|  | NCED3-N1(Chip)-R | TCGTGGCTCAAGAGCTTC |
|  | NCED3-N2(Chip)-F | CAACAAACGAGTCAACAGA |
|  | NCED3-N2(Chip)-R | CATAATGGAAGGTGTATAT |
| Gene expression | Actin-qPCR-F | CATCAGGAAGGACTTGTACGG |
|  | Actin-qPCR-R | GATGGACCTGACTCGTCATAC |
|  | HAT1-qPCR-F | ACAAAACCATCCTCTCCAGC |
|  | HAT1-qPCR-R | CCTCTTCCAAATCCACCGTT |
|  | HAT2-qPCR-F | AGACTCCCATGGAACCAAAC |
|  | HAT2-qPCR-R | CTCACTTCTCTTCCCGCTAATG |
|  | HAT3-qPCR-F | AGGACGAGACAAGTTGAAGTG |
|  | HAT3-qPCR-R | AGTGGGAGGTTTCATGTGC |
|  | ABA3-qPCR-F | AGTGGATATTGAAGAGGCAGC |
|  | ABA3-qPCR-R | CACCAGATCTAGATTAAACCTCAGG |
|  | NCED3-qPCR-F | GAGTGTCCTGTCTGAAATCCG |
|  | NCED3-qPCR-R | CGAATCCTGAGACTTTAGGCC |
|  | RD29A- qPCR-F | TCCAAAGTTACTGATCCCACC |
|  | RD29A- qPCR-R | CGAATCCTTACCGAGAACAGAG |
|  | RD22- qPCR-F | GTATTGTGCGACGTCTTTGG |
|  | RD22- qPCR-R | GCGAATGGGTACTTCTGTTTG |
|  | HAI1- qPCR-F | TCCTTACGCCGGAGAAAATC |
|  | HAI1- qPCR-R | TCGTGACATTGCAAGTACTCC |
|  | PP2CA- qPCR-F | GAAGACGAGACATGGAAGACG |
|  | PP2CA- qPCR-R | CTTAACCATCGTCTCTGTCCAC |
|  | HAI2- qPCR-F | GCCGTTGTCTCCGTCATTAC |
|  | HAI2- qPCR-R | GCACCGTCCCAGTATATCAC |
